# Implantable Biophotonic Device for Wirelessly Cancer Real‐Time Monitoring and Modulable Treatment

**DOI:** 10.1002/advs.202503778

**Published:** 2025-04-15

**Authors:** Renhao Nie, Qingyan Jia, Yuanying Li, Changhan Yan, Xiyin Liu, Yaolan Tao, Jianhong Zhang, Peng Li, Wei Huang

**Affiliations:** ^1^ State Key Laboratory of Flexible Electronics (LoFE) Frontiers Science Center for Flexible Electronics (FSCFE) Xi'an Institute of Flexible Electronics (IFE) and Xi'an Institute of Biomedical Materials & Engineering (IBME) Northwestern Polytechnical University 127 West Youyi Road Xi'an 710072 China; ^2^ School of Flexible Electronics (SoFE) and Henan Institute of Flexible Electronics (HIFE) Henan University 379 Mingli Road Zhengzhou 450046 China

**Keywords:** bioelectronic device, oxyhemoglobin saturation monitoring, photodynamic therapy, photoelectric sensor, tumor treatment

## Abstract

Miniaturized bio‐devices present a promising solution to address the limitations of conventional therapeutic equipment, such as bulkiness and high cost, while facilitating continuous monitoring of cancer development and treatment progression. Herein, a flexible wirelessly implantable biophotonic device is developed via integrating an oxyhemoglobin saturation (sO_2_) sensing probe, a low‐power Bluetooth microcontroller unit, and a wireless power module on a flexible printed circuit board. It is fabricated to identify tumors (˂ 30 mm^3^) from normal tissues by monitoring sO_2_ levels indicative of the tumor hypoxic microenvironment. Furthermore, this device can also evaluate the therapeutic progression of chemotherapy drugs like vadimezan, which reduces sO_2_ levels by disrupting tumor angiogenesis. The µ‐LED in the biophotonic device can function as a light source for in situ photodynamic therapy while simultaneously monitoring the oxygen consumption during the treatment process. The biophotonic device is lightweight, thin, and flexible, allowing seamless implantation within the body. It operates via wireless power and data transmission without disrupting normal physiological activities. Hence, the biophotonic device is capable of concurrently achieving precise tumor discrimination, modulable in situ treatments, and real‐time progression monitoring, enabling the evaluation and optimization of therapeutic efficacy.

## Graphical abstract

A wirelessly implantable biophotonic device is capable of distinguishing tumors and evaluating their progression by monitoring sO_2_ levels indicative of the tumor hypoxic microenvironment. Additionally, this device is not only employed for in situ PDT but also for concurrent monitoring of chemotherapy and PDT progress, thereby enabling the evaluation and optimization of therapeutic efficacy.

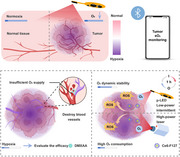

## 1 Introduction

In continuous and repetitive cancer treatments, it is imperative to concurrently monitor cancer progression throughout the course of treatment in order to evaluate and optimize therapeutic efficacy.^[^ ^1^ ^]^ Biophotonic technique enables the acquisition of biochemical and morphological insights into specific cells or tissues by leveraging a series of photophysical phenomena, including absorption, scattering, and emission that are initiated through the interaction between light and biological tissues.^[^ ^2^ ^]^ The development of diverse biophotonic devices has significantly contributed to the improvement of tumor diagnosis and therapy, even theranostics, owing to their multifunctionality, minimally invasiveness, and high spatial‐temporal selectivity.^[^ ^3^ ^]^ For instance, the optical biopsy device that integrates micro inorganic light‐emitting diodes (µ‐LED) and phototransistor has been proposed as an auxiliary surgical resection tool to effectively differentiate malignant tumor from normal tissues through measuring the amount of light scattered through the tumor.^[^ ^4^ ^]^ Simultaneously, the real‐time acquisition of in‐situ tumor‐related information could be achieved by the integration of wireless power and data transmissions.^[^ ^5^ ^]^

Photodynamic therapy (PDT) utilizes specific wavelength light to irradiate photosensitizers, converting O_2_ into highly biotoxic reactive oxygen species (ROS), thereby inducing oxidative damage to biomolecules such as proteins, lipids, and DNA in tumor cells.^[^ ^6^ ^]^ To meet the clinical requirements of portability, ease of operation, and light delivery for deep‐seated tumor treatment, implantable biophotonic devices utilizing wireless power to activate µ‐LEDs for on‐demand in situ PDT have been developed in recent years.^[^ ^7^ ^]^ In addition, the tumor size post‐PDT could be continuously monitored by incorporating the aforementioned optical biopsy strategy.^[^ ^8^ ^]^ These state‐of‐the‐art investigations present innovative insights for the development of a biophotonic technique that facilitates simultaneous real‐time tumor monitoring and customizable treatment, ultimately leading to efficacious anti‐cancer outcomes.

**Figure 1:**
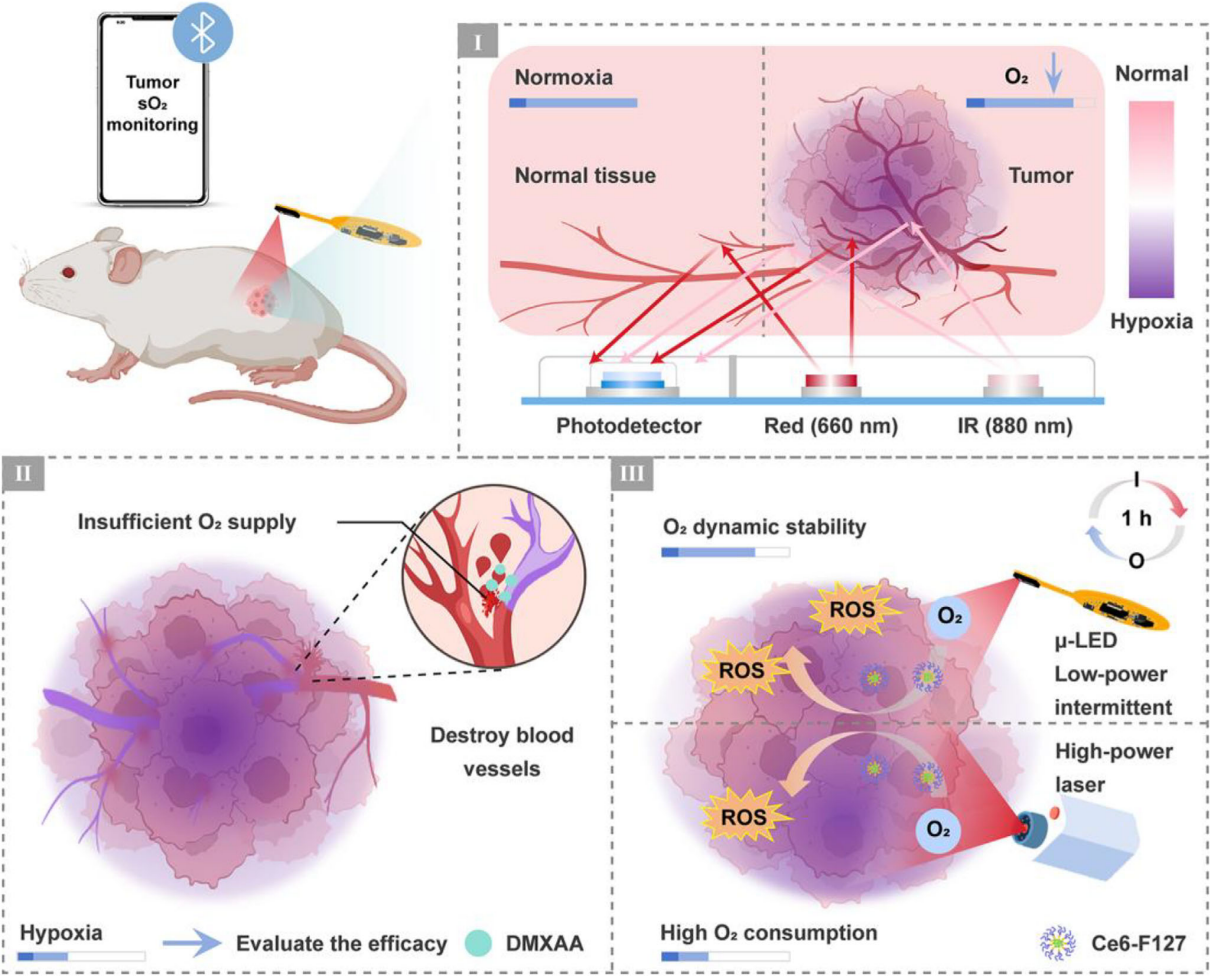
Schematic illustration of biophotonic device for real‐time cancer monitoring and modulable chemotherapy and PDT. I) Monitoring sO_2_ levels for distinguishing between normal tissue and tumors. II) Monitoring the vascular damage‐related chemotherapy process. III) In situ PDT of tumors.

The aberrant vascular structure of tumors leads to insufficient O_2_ supply, while the rapid proliferation of tumor cells results in an elevation in O_2_ consumption, thereby causing a reduction in sO_2_ levels within tumors compared to normal tissues.^[^ ^9^ ^]^ The real‐time sO_2_ monitoring can provide valuable insights into the growth and metabolism behaviors of tumors.^[^ ^10^ ^]^ In this work, we meticulously designed an implantable biophotonic device that integrates a sO_2_ sensing probe, a low‐power Bluetooth microcontroller unit (MCU), and a wireless power module on a flexible printed circuit board (FPCB). The biophotonic device is characterized by its light, thin, and flexible features, enabling it to be seamlessly implanted within the body and operated through wireless power and data transmissions from a cellular phone without interfering with normal physiological activities. Specifically, the sO_2_ levels of tumor can be accurately monitored, allowing for the detection of a 10%–15% reduction compared to normal tissues attributed to tumor hypoxic microenvironments, thus offering potential for specific monitoring of tumor growth status (**Figure** **1**I).

Certain chemotherapy drugs, such as vadimezan (DMXAA), primarily achieve their therapeutic objectives by disrupting tumor angiogenesis and impeding the delivery of essential nutrients and oxygen.^[^ ^11^ ^]^ The damage to tumor angiogenesis can also result in a reduction of sO_2_ levels.^[^ ^12^ ^]^ The biophotonic device facilitates the assessment of therapeutic progress and effectiveness of DMXAA in both intravenous and intra‐tumoral administrations (Figure 1II), offering valuable references for optimizing drug delivery routes, dosage, and administration timing. Meanwhile, the µ‐LED in the sO_2_ probe can serve as an internal light source to activate the photosensitizer Ce6‐F127, thereby generating a substantial amount of ROS at the tumor site (Figure 1III). This approach facilitates controlled and sustained in situ PDT, thus eliminating the need for a bulky external light source and its limitations on tissue penetration depth. Besides, the biophotonic device can also be used to monitor the O_2_ consumption during PDT. The high‐intensity light has been demonstrated to result in rapid O_2_ depletion, leading to suboptimal PDT efficacy; conversely, employing low‐power and intermittent irradiation from µ‐LEDs ensured adequate O_2_ supply, thereby enhancing the outcomes of PDT.

## 2 Results

### 2.1 Biophotonic Device for sO_2_ Monitoring

**Figure 2:**
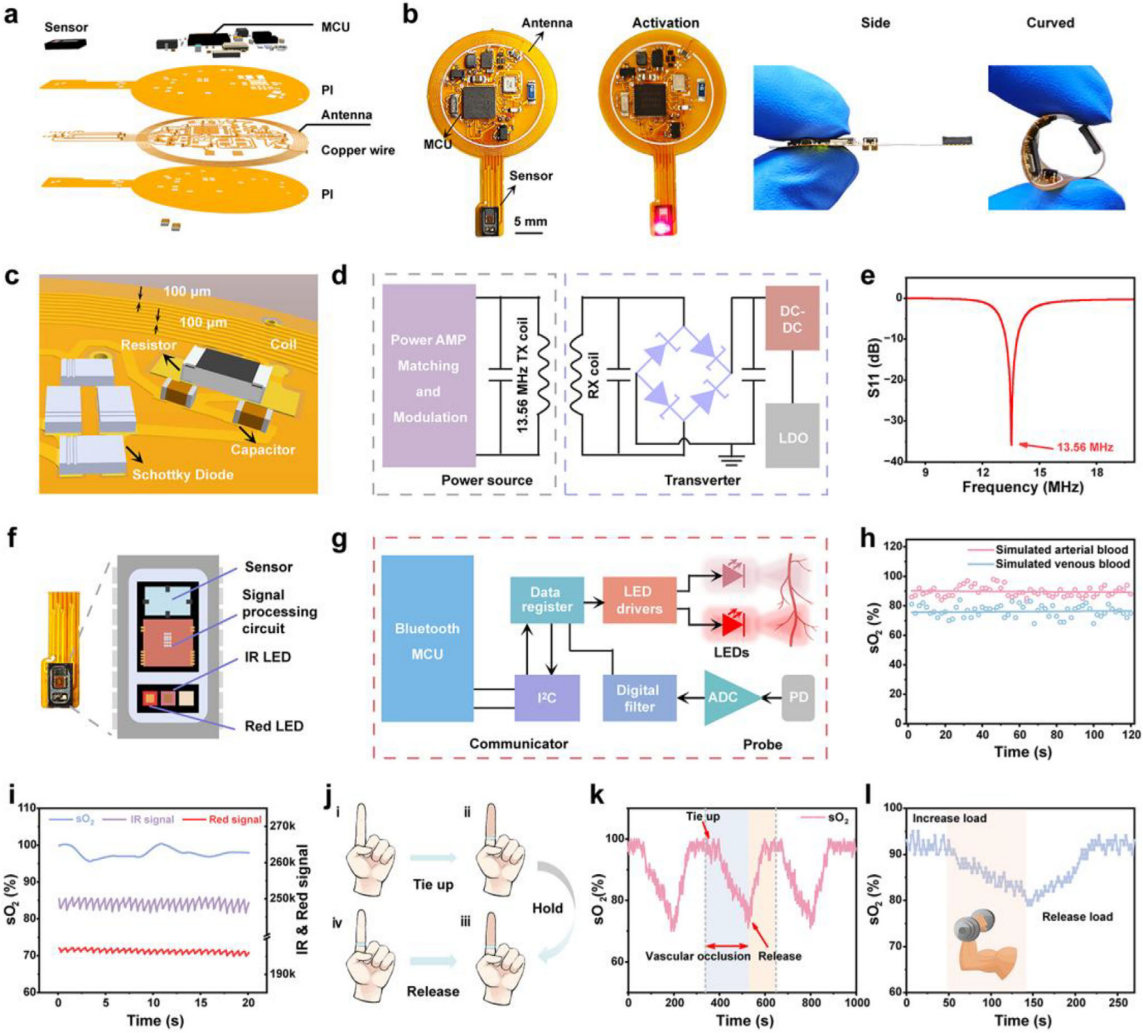
Fabrication and sO_2_ monitoring of Biophotonic device. a) Explosion diagram of biophotonic devices. b) Physical photos of biophotonic devices. 3D model diagram c) and principle schematic diagram d) of wireless power receiving antenna and resonant rectification circuit. e) Finite element simulation (FES) of the scattering parameter S11 for the wireless power receiving LC resonant circuit. 3D model diagram f) and schematic diagram g) of the sO_2_ sensing probe. sO_2_ detection of artificial blood h) and forearm normal skin tissue i). j) Flow chart of the bundle finger vein occlusion experiment. k) sO_2_ monitoring of fingertip during the process of occluding finger veins. l) sO_2_ monitoring of muscle tissue during arm weight‐bearing exercises.

The integrated biophotonic device is characterized by its compact size (featuring a circular main body with a radius of 12 mm and a sensing part extended by wires measuring 14 mm in length), lightweight design (weighing merely 370 mg, with a thickness of 1.1 mm), and exceptional flexibility (**Figure** **2**a,b; Figures S1–S4, Supporting Information). The biophotonic device consists of two functional modules: a thin resonant antenna and its power management module, which can be magnetically coupled with an external RF transmitter to collect RF energy and provide power for the device; and another module for sO_2_ acquisition, signal processing, and data transmission. The wireless power module includes an LCR resonant circuit, a rectification and filtering circuit, and a voltage stabilization circuit, with a resonant frequency of 13.56 MHz (Figure 2c–e; Figures S5 and S6, Supporting Information).^[^ ^13^ ^]^ The sO_2_ sensor utilizes the Max30102 sensing probe, which primarily comprises an LED driver, red (660 nm) and infrared (IR, 880 nm) LEDs, a photodetector, and a signal processing circuit (Figure 2f; Figure S7, Supporting Information). The sO_2_ sensor measures the attenuated light signals of hemoglobin and deoxyhemoglobin in the blood through reverse scattering, and stores the obtained photoelectric signals in a register after undergoing analog‐to‐digital conversion and filtering processing (Figure 2g). Subsequently, the MCU retrieves the raw data from the sensor's registers through I^2^C communication. After performing calculations and processing, it transmits the data to the user's mobile phone via Bluetooth for continuous monitoring of sO_2_. Further details regarding the components are provided in Table S1 (Supporting Information).

By configuring an artificial blood colloid, the ability of the biophotonic device to monitor sO_2_ was evaluated. A commercially available bovine hemoglobin powder, containing predominantly methemoglobin, in Phosphate‐buffered saline (PBS) solution (25 g L⁻¹), appears dark red in air, simulating venous blood.^[^ ^14^ ^]^ After adding an excess of reducing agent Na_2_S_2_O_4_ to the solution (in a mass ratio of 4:1 with the hemoglobin powder), the solution turned bright red, simulating arterial blood. The two solutions demonstrate distinct absorbance differences at wavelengths of 660 and 880 nm, a characteristic that serves as the foundation for sO_2_ measurements (Figure S8, Supporting Information). The sO_2_ levels observed in the two solutions showed a significant disparity; simulated arterial blood exhibited an sO_2_ level of ≈90%, whereas simulated venous blood displayed an sO_2_ level of ≈78% (Figure 2h).

The biophotonic device for assessing the sO_2_ level on the body surface underwent further evaluation. First, the device was applied to collect sO_2_ from normal skin tissue on the forearm, where its values remained consistently within the range of 95–100% (Figure 2i). Subsequently, a finger venous occlusion experiment was conducted to simulate hypoxia in the limb. The blood flow at the base of the index finger was restricted using a rubber band while attaching the sO_2_ probe to its tip. The occlusion lasted for 3 min. During this process, the finger exhibited a dark red hue and the sO_2_ gradually declined to ≈75%. After the release of occlusion, the device detected a rapid increase in sO_2_ values, followed by stabilization at pre‐occlusion levels (98%) (Figure 2j,k). To achieve the O_2_ consumption in human muscle, participants engaged in isometric exercises by lifting weights with their arms. The sO_2_ levels gradually decreased as muscle O_2_ consumption increased during exercise, but returned to normal levels (≈92%) after releasing the load (Figure 2l). In addition, we performed a comprehensive series of tests to compare the measurement accuracy of our device with that of commercially available sO_2_ testing equipment (Xiaomi smartwatch) on human and mouse skin. According to the experimental results, it is clear that the accuracy and stability of sO_2_ measurements obtained by our device are closely consistent with those of the commercial equipment under various conditions (Figure S9, Supporting Information). The aforementioned experiments have conclusively demonstrated the exceptional operational stability and commercial accuracy of the biophotonic device in testing sO_2_ levels.

### 2.2 In Vivo Implantation of Biophotonic Device

**Figure 3:**
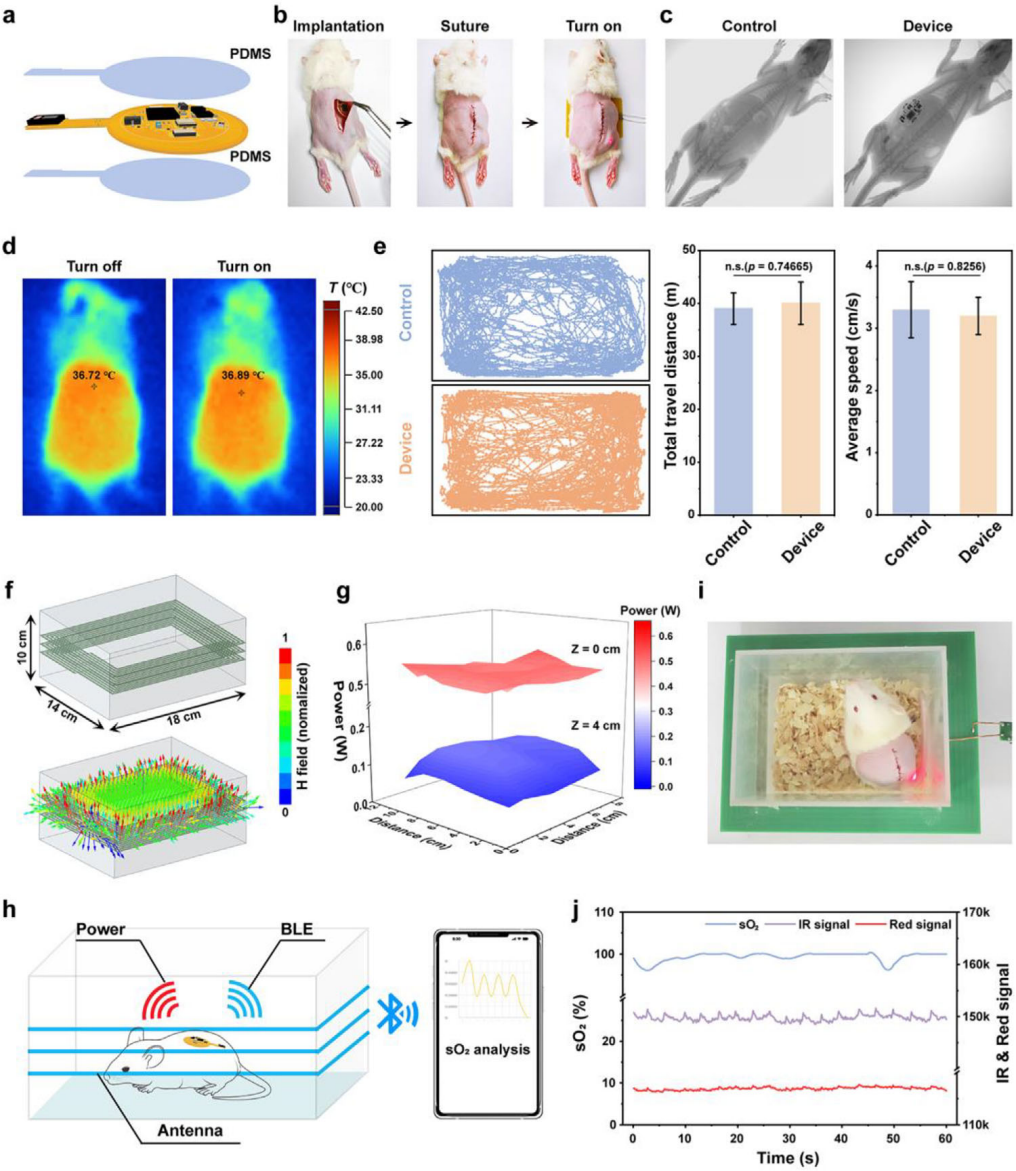
In vivo implantation and performance investigation of the biophotonic device. a) Schematic diagram of PDMS‐packaged biophotonic device. b) Surgical photos of subcutaneous implantation of the biophotonic device in mice. c) X‐ray image of biophotonic device implanted in mice. d) Infrared images of biophotonic devices operating subcutaneously in mice at 0 and 300 s. e) Behavior trajectory analysis of mice implanted with biophotonic device. f) 3D model of a WPT transmitter and its FES of the magnetic field. g) The power collection of the RX coil at distances of 0 cm and 4 cm along the Z‐axis from the center of TX coil. h) Schematic of sO_2_ monitoring in mice implanted with biophotonic device within the WPT transmitter. The image of mice i) and the sO_2_ data j) obtained from implanted biophotonic device.

Polydimethylsiloxane (PDMS) exhibits superior flexibility and optical transparency, which ensures efficient light transmission from µ‐LEDs while offering robust protection for electronic components. The biophotonic device was encapsulated with PDMS to prevent infiltration of biological fluids into electronic components (**Figure** **3**a; Figure S10 and Video S1, Supporting Information).^[^ ^15^ ^]^ The device could maintain stable operation in water for an extended period of time under the protection of PDMS coating (Figure S11, Supporting Information). The device was implanted into rats and positioned beneath them on a compact wireless power transmission (WPT) board measuring 5.5 cm × 4 cm in length and width. It was observed that the device maintains consistent operational stability following full subcutaneous implantation in rats (Figure 3b). The X‐ray image of a subject implanted with the device revealed its attachment to the rat's back while positioning the sensor at the predetermined tumor site (Figure 3c). The temperature rise of the device during normal operation was observed to be below 3 °C, thereby ensuring its safe functionality without causing thermal damage to healthy tissues (Figure 3d). The movement behavior of mice was analyzed by utilizing cameras and extracting feature trajectories using deep neural networks, in order to evaluate the impact of implanted devices. The behavioral trajectory following device implantation exhibited a similarity to that of the control mice (Figure 3e). Consequently, the influence of device implantation on mouse walking distance and speed could be deemed negligible.

The construction of an external WPT module using an LCR resonant circuit was implemented to ensure stable operation of the biophotonic device during normal mouse movement after subcutaneous implantation. The RF transmitting end (TX) consists of a vertically arranged cage structure comprised of three layers of coils. The FES results illustrated the distribution of magnetic field in the TX coil,^[^ ^16^ ^]^ as depicted in Figure 3f. The magnetic field strength in the horizontal direction of the middle layer TX coil exhibited a gradual increase from the center toward its surroundings, while in the vertical direction, it gradually decreased from the center to its surrounding areas (Figure S12, Supporting Information). The resonant frequency matching between the TX and RX coils was subsequently analyzed using FES (Figure S13, Supporting Information). The resonance peaks of the loss parameters (S_(RX, RX)_ and S_(TX, TX)_) and gain parameter (S_(TX, RX)_) exhibited a consistent frequency of 13.56 MHz, thereby ensuring optimal theoretical energy transfer efficiency (Figure S14, Supporting Information). The wireless energy transmission efficiency of the RX coil was subsequently analyzed using FES at various vertical distances (Z) from the center of the TX coil, while testing the received power values of the device at different positions within the TX coil. The power transmission efficiency gradually decreases as the vertical distance Z increases from 0 to 5 cm, which can be attributed to the diminishing gain parameters S_(TX, RX)_ with increasing vertical distance Z (Figure S15, Supporting Information). However, even at a 5 cm distance between the RX coil and TX coil, the device still receives over 60 mW of power (Figure 3g), which exceeds the power consumption of the nRF52832 chip in low‐power Bluetooth mode (≈ 17 mW). In addition, we conducted tests on the current values received by the power supply section of the device at various angles within a wireless power transmission cage. As shown in Figure S16a (Supporting Information), it can be observed that even at an angle of 90 degrees, the power module of the device continues to receive a current of 20 mA, thereby satisfying the current requirement for the proper functioning of the nRF52832 chip (13 mA). Meanwhile, we have carried out experiments to investigate the wireless power transmission performance of the device embedded in tissues of varying thicknesses (pig skin). It can be observed that biological tissues exert only a minimal effect on wireless power transmission (Figure S16b,c, Supporting Information). Even with a tissue thickness of 8 cm, the current received by the wireless power management module of the device decreases by ≈6%, which is insufficient to interfere with the normal operation of the device. Therefore, the receiving coil positioned within the magnetic field emission cage can reliably obtain energy at any position to power the device, thereby providing a wide and effective wireless power supply distance (Figure S17, Supporting Information). The implanted biophotonic device, based on the aforementioned wireless energy transmission system, facilitated continuous and stable monitoring of sO_2_ in the skin during mouse movement (Figure 3h–j and Video S2, Supporting Information).

### 2.3 Tumor sO_2_ Monitoring of Biophotonic Device

**Figure 4:**
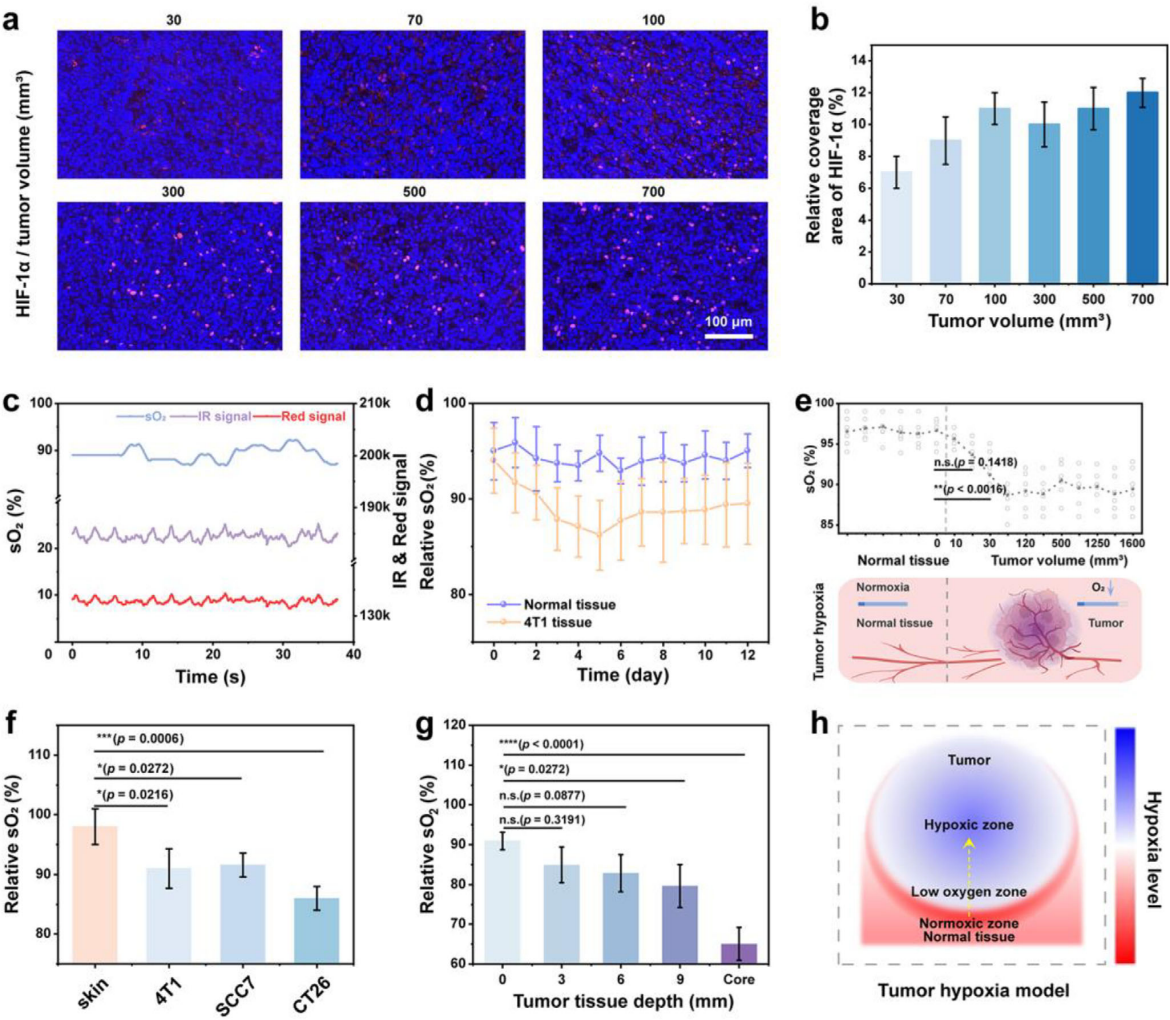
sO_2_ monitoring of tumor via biophotonic device. HIF‐1α staining images a) and quantitative statistical graphs b) of tumors with varying sizes. c) sO₂ curve of tumor detected by biophotonic device. d) sO₂ levels of tumor throughout its growth process with recording initiated at a volume of ≈10 mm^3^. e) Comparison of sO_2_ levels between tumors at different growth stages and normal tissues. f) sO_2_ values for different types of tumors at a volume of 300 mm^3^. g) sO₂ levels in tumor at various depths. h) Simulated hypoxia levels vary with tumor depth.

A murine model with 4T1 tumor xenografts was established to investigate the alterations in sO_2_ levels across varying tumor volumes. The hypoxia status of tumors at different growth stages was first assessed by means of HIF‐1α immunofluorescence staining. The hypoxia level in tumor tissue gradually increases with the increase in tumor volume during the early stage of tumor growth, as depicted in **Figure** **4**a,b, when the tumor volume is less than ≈100 mm^3^. However, as the tumor volume continues to grow larger, the hypoxia level in tumor tissue tends to stabilize. The experimental results demonstrated a consistent pattern when the biophotonic device was utilized to examine the sO_2_ levels of tumors at different stages of growth (Figure 4c–e; Figure S18, Supporting Information). The sO_2_ level of tumor tissues exhibited a reduction of ≈10% in comparison to that of healthy skin tissues. The increase in tumor volume during growth was accompanied by a slight initial decrease in sO_2_ levels, followed by a gradual stabilization. This initially rapid decrease in sO_2_ levels may be attributed to the swift exhaustion of nutrients during early tumor growth, which occurs before the establishment of fully developed surrounding blood vessels. However, the sO_2_ levels gradually stabilize as blood vessels form, yet they still remain lower than those observed in normal tissues.

The SCC7‐ and CT26‐tumor bearing mouse models were also established. The sO_2_ levels in tumor were found to be ≈10%–15% lower than those in normal skin tissue, with variations observed among different types of tumors (Figure 4f). It is speculated that the sO_2_ level of tumor reflects its heterogeneous metabolic characteristics, wherein the aberrant proliferation of cancer cells results in heightened O_2_ consumption and consequently reduces the sO_2_ level within the tumor. Additionally, the variations in sO_2_ levels may be attributed to disparities in tumor growth rates and varying degrees of vascular defects. In addition, the sO_2_ levels were assessed at various depths within the tumor. As depicted in Figure 4g,h, with the increasing depth of the tumor, a gradual decrease in sO_2_ levels was observed. This phenomenon is closely associated with the heterogeneity and metabolic characteristics of the tumor microenvironment. The insufficient delivery of O_2_ to deeper regions of the tumor results in variations in sO_2_ levels, which are characterized by gradient transitions from the normoxic zone, through the hypoxic zone, to the severely hypoxic zone.

### 2.4 Tumor Vascular Disruption and sO_2_ Monitoring

**Figure 5:**
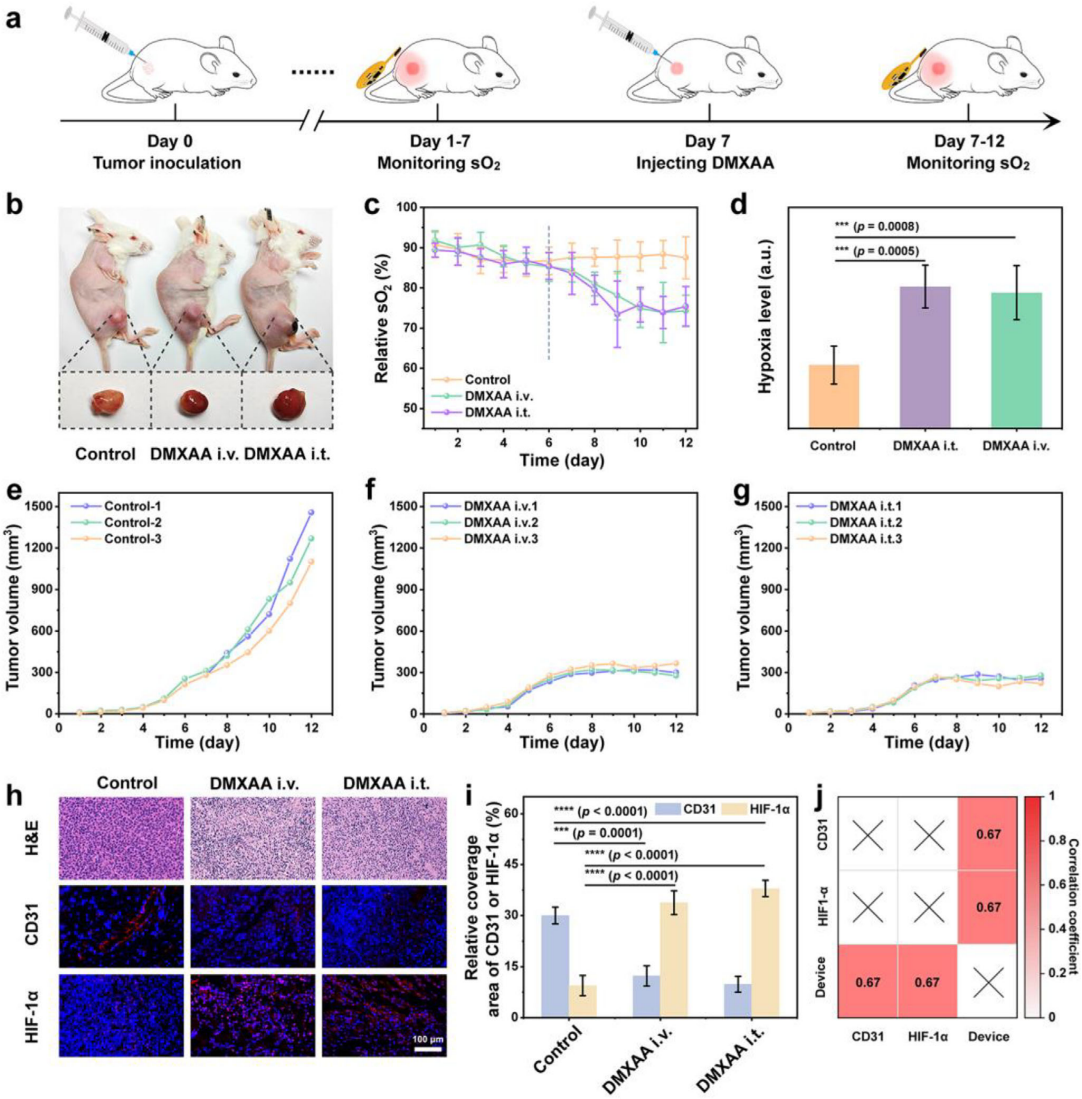
sO_2_ detection for monitoring tumor vascular disruption and modulable chemotherapy. a) Diagram of the experimental protocol for the tumor vascular destruction model. b) Photos of tumors following vascular destruction. c) sO_2_ levels of tumor before and after vascular destruction. d) Hypoxia levels of tumor before and after vascular destruction. e–g) Growth curves of tumors after different treatments. h) H&E, CD31, and HIF‐1α staining images of tumors after different treatments. i) Quantitative statistical analysis of CD31 and HIF‐1α staining images. j) Correlation analysis between CD31 and HIF‐1α indicators and tumor hypoxia levels measured by biophotonic device.

From a clinical perspective, certain chemotherapy drugs, such as DMXAA, exert their therapeutic effects by disrupting tumor angiogenesis and impeding nutrient supply. The damage to blood vessels will further result in a reduction of tumor sO_2_ levels, thereby facilitating the monitoring of chemotherapy process using our designed biophotonic devices. The administration of DMXAA was performed via intravenous and intratumoral routes in 4T1 tumor‐bearing mice, respectively. In comparison to the non‐injected group, the alterations in sO_2_ levels were assessed following tumor vascular disruption (**Figure** **5**a). After a 24 h intravenous or intratumoral administration of DMXAA, the treated tumors exhibited obvious bleeding symptoms and acquired a deep red hue, while the control group maintained a pink coloration (Figure 5b; Figure S19, Supporting Information). Meanwhile, the biophotonic devices were utilized to examine the alterations in sO_2_ levels subsequent to blood vessel disruption. As expected, compared to the control group, the sO_2_ levels in tumor exhibited a significant decrease of ≈10% following DMXAA injection, indicating a notable increase in tumor hypoxia (Figure 5c,d). Additionally, the tumor growth was significantly impeded due to vascular disruption‐induced insufficiency of O_2_ and nutrient supply (Figure 5e–g). The H&E staining also revealed an augmented occurrence of cellular apoptosis following DMXAA treatment (Figure 5h).

The immunofluorescent staining of CD31, an endothelial marker, was employed to evaluate the changes in the tumor vascular system subsequent to DMXAA treatment. As depicted in Figure 5h,i, the tumor sections from the DMXAA treatment group exhibited a discontinuous distribution of red fluorescent CD31 staining, indicating significant disruption of tumor vascular integrity. In contrast, the control group maintained nearly intact tumor vasculature. The endogenous marker HIF‐1α serves as an indicator to gauge the extent of hypoxia within the tumor. The control group exhibited only a slight presence of HIF‐1α positive red fluorescence; in contrast, following the administration of DMXAA, there was a dense distribution of red fluorescence indicating a significant hypoxic response subsequent to vascular damage (Figure 5h,i). The above experimental results demonstrated an intensified disruption of tumor vasculature and an increased tissue hypoxia following DMXAA injection, which is consistent with the measured sO_2_ values through the biophotonic device (Figure 5j). Therefore, the device can be utilized for monitoring the treatment progress of drugs that damage blood vessels, thereby offering potential for guiding and optimizing medication.

### 2.5 Photodynamic Therapy and sO_2_ Monitoring

The biophotonic device incorporates µ‐LEDs emitting at wavelengths of 660 and 880 nm, which serve not only as sO_2_ monitoring probes but also as light sources for PDT. The Ce6 molecule, which exhibits an absorption peak at 660 nm, was selected as the photosensitizer to match with 660 nm µ‐LEDs. To enhance the limited water solubility, F127 was utilized for the nanosizing of Ce6 (Figure S20a, Supporting Information). The successful preparation of Ce6‐F127 was confirmed through transmission electron microscopy (TEM), dynamic light scattering (DLS), and UV–vis absorption spectroscopy (Figure S20b–d, Supporting Information). The Ce6‐F127 nanoparticles were observed to exhibit excellent dispersion, displaying a quasi‐spherical shape with a hydrated particle size of ≈200 nm, and showed significant absorption peaks at 410 and 660 nm corresponding to the presence of Ce6. Under the illumination of 660 nm µ‐LED, the Ce6‐F127 demonstrated remarkable photocatalytic ROS generation capability, leading to a significant reduction in the intensity of 1,3‐diphenylisobenzofuran (DPBF)’s characteristic absorption peak at 417 nm (Figure S21, Supporting Information). Additionally, the alamarBlue and LIVE/DEAD staining experiments demonstrated that Ce6‐F127 achieved a kill rate exceeding 95% in 4T1 cells when exposed to light, while minimal cell death was observed in both the light‐only group and the Ce6‐F127 non‐light group (Figure S22, Supporting Information). The flow cytometry analysis proved that PDT triggers cell apoptosis through the generation of ROS, with the Q2 and Q3 regions corresponding to early and late apoptosis, respectively (Figure S23, Supporting Information).

**Figure 6:**
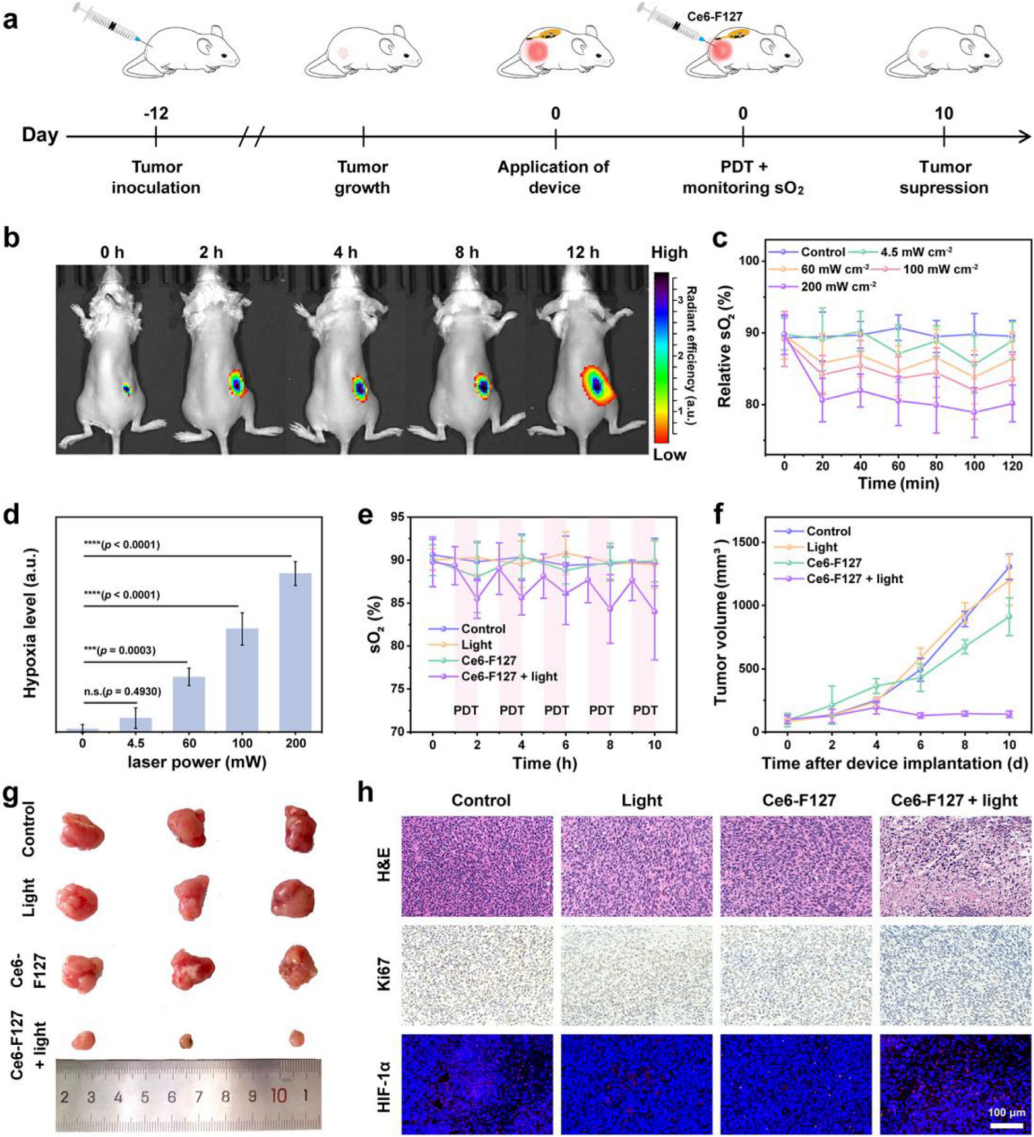
In situ modulable PDT and its process monitoring. a) Flowchart of the animal experimental model for PDT. b) Metabolic imaging of Ce6‐F127 in tumors after intratumoral injection. sO_2_ levels c) and hypoxia levels d) of tumor tissues during intermittent irradiation at every 20 min intervals with different laser powers. e) sO_2_ levels of tumor tissues under various intermittent hourly treatment regimens. f) Temporal profile of tumor volume during the 10‐day treatment period. g) Photographs of tumors on the 10th day following different treatments. h) H&E‐, Ki67, and HIF‐1α‐staining images of tumors on the 10th day following different treatments.

The therapeutic effect of the biophotonic device on 4T1 tumor‐bearing mice was subsequently assessed (**Figure** **6**a). Following intratumoral administration of Ce6‐F127, the distribution of photosensitizers within mouse tumors and major organs including the heart, liver, spleen, lungs, and kidneys was monitored using a small animal imaging system. Remarkably, Ce6‐F127 exhibited a prolonged retention period within the tumor site (Figure 6b; Figure S24, Supporting Information). The impact of varying laser powers on O_2_ consumption during the process of PDT was investigated by assessing tumor sO_2_ levels after 20 min of light exposure and 20 min of darkness for three cycles, utilizing power densities ranging from 4.5 to 200 mW cm^−2^. The observation revealed that under low light power irradiation, there is a slight decrease in tumor sO_2_, which promptly recovers upon cessation of light exposure. Conversely, high laser power induces rapid reduction in tumor sO_2_ with more challenging recovery (Figure 6c,d). Subsequently, we conducted a comparison of the cytotoxic effects of PDT on 4T1 cells under identical total illumination energy conditions (power × time), utilizing both a high‐intensity laser (100 mW cm⁻^2^) and a low‐intensity LED (4.5 mW cm⁻^2^). As shown in Figure S25a (Supporting Information), upon 5 min of illumination, the laser successfully eradicated 71.5% of the 4T1 cells. However, extending the illumination time beyond this duration did not yield improved therapeutic outcomes. To exclude the potential photobleaching effect of the laser on Ce6, we systematically evaluated its absorption spectra following various illumination durations (Figure S25b, Supporting Information). While a portion of Ce6 underwent photobleaching, ≈56% remained intact after 30 min of continuous illumination. Consequently, we demonstrated that the primary reason for the lack of therapeutic effects in cells illuminated for 5 to 30 min was the rapid depletion of O_2_ under high‐intensity light irradiation, coupled with an inability to replenish it promptly. However, under LED illumination, despite an initially less pronounced effect compared to laser treatment, the therapeutic outcome surpassed that of the laser when the illumination duration was extended to 8 and 11 h, which corresponded to 20 and 30 min of laser exposure, respectively (Figure S25c, Supporting Information). Therefore, a low‐power and intermittent light exposure is more beneficial for maintaining high O_2_ levels in tumors to ensure the effectiveness of PDT.

The biophotonic device was employed for intermittent light exposure, with a duration of 1 h every 2 h, and a total of 5 cycles were implemented for PDT (Figure S26, Supporting Information). The sO_2_ monitoring results demonstrated that as the irradiation time was prolonged, there was only a marginal 5% reduction in the tumor's sO_2_ level (Figure 6e). In addition, the tumor growth rates exhibited notable variations across the various treatment groups. The tumors in the PBS, light‐only, and Ce6‐F127 groups showed continuous growth; however, tumor growth was significantly suppressed subsequent to the administration of Ce6‐F127 followed by light irradiation (Figure 6f,g). The effectiveness of PDT was further validated by the positive results obtained from H&E and Ki‐67 staining.^[^ ^17^ ^]^ The PDT‐treated group exhibited significantly higher levels of tumor cell death compared to the control groups, effectively suppressing tumor growth and proliferation (Figure 6h; Figure S27, Supporting Information). The results of HIF‐1α staining indicated that, compared to the control, light‐only, and Ce6‐F127 groups, the red fluorescence intensity in the PDT group exhibited only a slight increase. This suggests that hypoxia was not significantly exacerbated, which is consistent with the observed decrease in tumor sO_2_ levels as measured by the device. Therefore, the biophotonic device not only facilitates in situ PDT by eliminating the necessity for a cumbersome external light source and overcoming its limitations on tissue penetration depth, but also enables real‐time monitoring of O_2_ consumption during PDT, ensuring an adequate oxygen supply to enhance the efficacy of PDT.

We conducted a comprehensive evaluation of the biocompatibility and immune response of the implanted device in mice. This assessment was performed using H&E staining, Masson trichrome staining, and immunofluorescence staining for M1 macrophages (CD68) and M2 macrophages (CD206). As shown in Figure S28 (Supporting Information), on the 5th day post‐implantation, prominent inflammatory reactions attributable to the surgical procedure were observed. However, by the 15th day, no significant inflammatory responses or tissue fibrosis were detected, suggesting that the low stiffness and excellent biocompatibility of the implanted device effectively minimize rejection reactions to the greatest extent possible.^[^ ^18^ ^]^

## 3 Conclusion

Table S2 (Supporting Information) provides a comparative analysis of the characteristics of our biophotonic device related to those documented in prior studies on tumor monitoring and therapy. The reported devices have predominantly concentrated on either monitoring or treatment, featuring a single function without integration of both capabilities. Unlike the reported devices that rely on principles of light diffuse reflection, refraction, and molecular fluorescence, our biophotonic device distinguishes tumors from normal tissues and evaluates tumor progression by detecting sO_2_ levels indicative of the tumor hypoxic microenvironment. This proof of concept has been validated across various cancer models and demonstrates the capability to detect tumors smaller than 30 mm^3^ in volume. Furthermore, the microscale device is utilized not only for tumor treatments such as chemotherapy and PDT but also for concurrently monitoring treatment progression throughout the entire process, thereby enabling the evaluation and optimization of therapeutic efficacy.

In particular, our device is specifically designed for minimally invasive implantation in tumors that are either inoperable or challenging to resect completely, as well as for the prevention and management of tumor recurrence following surgical intervention. In clinical applications, physicians will be able to promptly acquire tumor information through this device, thereby enabling them to seize optimal treatment opportunities. Currently, the device we have developed is only suitable for treating mouse tumors. To address larger and more complex tumors, an array‐type µ‐LED patch must be engineered to meet the requirement for extensive coverage. Additionally, for the effective eradication of deep‐seated tumor cells, a photosensitizer compatible with the 880 nm wavelength µ‐LED should be designed. Furthermore, the long‐term biosafety of this device, for several months or even years, and the removal through secondary surgery are major obstacles that must be overcome before clinical application. Prospectively, an integrated implantable bioelectronic device with a significantly reduced footprint and enhanced responsiveness to the characteristics of tumor microenvironment would be highly advantageous for the tumor monitoring and treatment at a smaller region or even at the cellular scale. This biophotonic device exhibits extensive applicability in the management of refractory and recurrent tumors, and is expected to transform the prevailing medical paradigm in clinical oncology.

## Conflict of Interest

The authors declare no conflict of interest.

## Author Contributions

W.H., P.L., and Q.J. proposed the conception of the project. R.N. and Q.J. performed the experiments and collected the data. Y.L., C.Y., X.L., Y.T., and J.Z. helped data analysis. R.N. and Q.J. prepared the manuscript. W.H. and P.L. revised the manuscript. All the authors discussed the results and commented on the manuscript.

## Supporting information



Supporting Information

Supplemental Video 1

Supplemental Video 2

## Data Availability

The data that support the findings of this study are available from the corresponding author upon reasonable request.
