# Supplementary material for: Implantable Biophotonic Device for Wirelessly Cancer Real‐Time Monitoring and Modulable Treatment
Source: Adv Sci (Weinh). 2025 Apr 15;12(26):2503778. doi: 10.1002/advs.202503778 (PMC12245013; doi:10.1002/advs.202503778)
Supplement: Supplementary file 1 — Supporting Information [file ADVS-12-2503778-s001.docx]

Supporting Information

**Implantable biophotonic device for wirelessly cancer real-time monitoring and modulable treatment**

*Renhao Nie,^a^ Qingyan Jia,*^a^ Yuanying Li,^a^ Changhan Yan,^a^ Xiyin Liu,^a^ Yaolan Tao,^a^ Jianhong Zhang,^a^ Peng Li,*^a,b^ Wei Huang*^a,b^*

^a^State Key Laboratory of Flexible Electronics (LoFE), Frontiers Science Center for Flexible Electronics (FSCFE), Xi’an Institute of Flexible Electronics (IFE) and Xi’an Institute of Biomedical Materials & Engineering (IBME), Northwestern Polytechnical University, 127 West Youyi Road, Xi'an 710072, China

^b^School of Flexible Electronics (SoFE) and Henan Institute of Flexible Electronics (HIFE), Henan University, 379 Mingli Road, Zhengzhou 450046, China

***E-mail:** [vc@nwpu.edu.cn](mailto:vc@nwpu.edu.cn); [iampli@nwpu.edu.cn](mailto:iampli@nwpu.edu.cn); [iamqyjia@nwpu.edu.cn](mailto:iamqyjia@nwpu.edu.cn)

**This PDF file includes:**

Experimental Section

Figures S1 to S23

Tables S1 to S2

Legends for videos S1 to S2

References (19 to 31)

**Other Supplementary Materials for this manuscript include the following:**

Videos S1 to S2

**Experimental Section**

**Materials:**

Dimethylsulfoxide (DMSO), PDMS, PBS and DPBF were obtained from Adamas. Trypsin, Penicillin-streptomycin solution and Dulbecco’s modified Eagle’s medium (DMEM) was purchased from Xi'an Jinbaoshun Biotechnology Co., Ltd. Alamar blue assay kits and LIVE/DEAD viability/cytotoxicity were obtained from Thermo Fisher Scientific (USA). Mouse breast cancer cells (4T1), colon cancer cells (CT26) and squamous cell carcinoma cells (SCC7) were ordered from Wuhan Servicebio Biotechnology Co., Ltd.

**Characterizations:**

The waveform generator (33500B series, Keysight Technologies,) and the RF power amplifier (ATA-8202, Aigtek) were used in the wireless power supply system. The LC resonant frequency was recorded with a Vector Network Analyzer (SV4401A, SYSJOINT). The voltage and current signals were measured with a digital multimeter (Fluke, USA). The animal behavior trajectory curve was acquired through the Animal Behavior Trajectory Video Analysis System V3.0 (Beijing Zhongshidi Chuang Technology Development Co., Ltd, China). The X-ray image was captured using an X-ray dynamic flat panel detector NDT0505J (IRAY technology, China). Thermal imaging data were captured utilizing a TiS10 thermal camera (Fluke, USA). TEM was performed using an FEI Talos F200X (Thermo fisher Scientific, USA). The UV−vis spectra were acquired on a UV-3010 spectrophotometer (Hi-tachi, Japan). The particle size of the photosensitizer assembly was measured using a zetasizer nano ZS Zen 3600 (Malvern, Britain).

**Design and manufacturing of electronic modules:**

The primary component of the biophotonic device is a FPCB composed with Cu/PI/Cu layers (18 μm/25 μm/18 μm), which was manufactured by Shenzhen Chuangxing Quick Technology Co., Ltd. using printed circuit board etching technology. The components are precisely mounted in the designated positions on the FPCB surface through SMT technology, establishing reliable electrical and mechanical connections with the FPCB via reflow soldering.

The components include: microcontroller (NRF52832, Nordic Semiconductor), blood oxygen sensor (Max30102, Maxim), power shielding inductor (FTC252012S100MBCA, cjiang), schottky diode (PMEG3010AESBYL, Nexperia), DC-DC voltage regulator chip (TPS62177, Texas Instruments), low-dropout regulators (DS8651-33S3 and DS8651-18SS3, DSTECH), crystal oscillator (X322532MOB4SI and X321532768KGD2SI, YXC), as well as resistors and capacitors. The low-power Bluetooth microcontroller (NRF52832) is programmed in C using the Keil V5 IDE for data sampling, processing, and Bluetooth communication.

The peripheral resonant coil of the device comprises 9 turns of 100 μm wide copper wire with a 100 μm spacing between turns, achieving an inductance of 3.59 µH and a quality factor of 15. The LCR resonant circuit, which consists of inductors, capacitors, and resistors, is designed to match the resonant frequency of 13.56 MHz of the external RF transmission coil. The full-wave rectifier bridge converts the AC voltage induced in the coil into DC voltage with the appropriate polarity. A ceramic capacitor is subsequently connected to the circuit to smooth the output waveform. The DC-DC voltage regulator chip TPS62177 is utilized to regulate the power supply voltage to a precise 3.3 V, while the low-dropout linear voltage regulator DS8651-33S3 ensures a stable power supply by providing an excellent power supply rejection ratio. The DS8561-18SS3 linear voltage regulator chip provides a stable 1.8V logic power supply to the blood oxygen sensing module.

**Electromagnetic simulation:**

The FES of electromagnetic field was conducted utilizing Ansys HFSS 2023 to ascertain the coil characteristics, scattering parameters, and power transmission efficiency within the resonant circuit. The RX coil was placed at different heights along the central axis of the TX RF cage. The magnetic field distribution within the RF transmission coil was analyzed utilizing Ansys Maxwell 2023. The simulation employed the default adaptive convergence criterion and utilized a 500 mm cubic domain as the radiation boundary condition to ensure computational accuracy.

**RF power transmission characteristics:**

The external wireless RF transmission power supply module employs LCR resonant circuits for efficient energy transfer. We fabricated a compact etched printed circuit board measuring 5 × 4 cm, integrated with an RF transmission antenna. The board features a line width and spacing of 0.5 mm, comprising 8 turns. Furthermore, we constructed an RF transmission cage measuring 18 × 14 × 10 cm, featuring three layers of coils. Each layer comprises 5 turns with a line width and spacing of 2 mm. The device was subjected to a curvature radius of 15 mm and subsequently mounted on an RF transmission board to assess its operational stability under bending conditions. To assess the performance of wireless power acquisition, the biophotonic device was positioned at various heights along the central axis of the RF transmission cage, and the load current was measured using a multimeter. The power management module of the biophotonic device is interfaced with a 1 kΩ resistive load, and the TX RF transmitter delivers an output power of 4 W. Furthermore, the biophotonic device was positioned at two distinct heights (0 cm and 4 cm) relative to the center of the RF transmission cage to assess its wireless power acquisition performance at varying locations.

**The principle of sO_2_ acquisition:**

The sO_2_ represents the ratio of oxygenated hemoglobin (HbO_2_) to the total hemoglobin in the blood, which can be quantified using the following formula:

$$sO_{2}=\frac{C[\mathrm{Hb}O_{2}]}{C\left[ \mathrm{Hb}O_{2} \right]+C\left[ \mathrm{RHb} \right]}\times100\%$$

where C[HbO_2_] and C[RHb] denote the concentrations of HbO_2_ and deoxygenated hemoglobin (RHb), respectively.

The Beer-Lambert law elucidates the relationship between light attenuation and the absorptive characteristics of the material through which it propagates.

$$I=I_{0}e^{-\varepsilon(\lambda)Cd}$$

Based on the formula, we can calculate that:

$$A=ln(\frac{I_{0}}{I})=\varepsilon(\lambda)Cd$$

where A is the attenuation, I_0_ is the incident light intensity, I is the received light intensity, $\varepsilon(\lambda)$ is the molar extinction coefficient, C is the concentration of material, and d is the optical path length.

The disparity in light absorption at 660 nm and 880 nm between arterial and venous blood constitutes the fundamental principle underlying sO_2_ measurement. The photoelectric blood oxygen sensor emits light at two distinct wavelengths into the tissue, detects the diffusely reflected light using a photodiode, and calculates the sO_2_ based on the degree of light attenuation. In the actual sO_2_ measurement process, light is also absorbed by various tissues, including bone, muscle, and fat. The observed light attenuation results from the cumulative absorption effects of these tissues. Consequently, the Beer-Lambert law can be further elaborated as follows:

$$A=d[\varepsilon_{{HbO}_{2}}\left( \lambda\right)C\left[ {HbO}_{2} \right]+\varepsilon_{RHb}\left( \lambda\right)C\left[ RHb \right]+\varepsilon_{other}\left( \lambda\right)C\left[ other \right]]$$

The Beer-Lambert law facilitates the measurement of sO_2_ by utilizing the molar extinction coefficients of HbO_2_ and RHb.

**Calculation of sO_2_:**

The MAX30102 employs two μ-LEDs with different wavelengths to determine the sO_2_ level by measuring the light absorption of HbO_2_ and RHb, operating with a sampling rate of 100 Hz. Normalization is essential for ensuring accurate and reliable calculations, given the varying amplitudes of the DC and AC components in the two-wavelength optical signals acquired by the sensor. To conduct subsequent calculations, a ratio parameter “R”, which is correlated with sO_2_, was established. Once the R value has been determined, curve fitting techniques or lookup table methods can be employed to ascertain the sO_2_ value. In the practical implementation, R was calibrated utilizing empirical data gathered from a substantial number of participants. Following the formula derivation and empirical adjustment, Maxim Integrated Products presents the subsequent equation for the best-fitting curve:

$$sO_{2}=aR^{2}+bR+c$$

where a, b, and c represent the calibration coefficients with values of a = -45.060, b = 30.354, and c = 94.845 respectively.

R represents the ratio of the AC component (𝐼_𝐴𝐶_, representing the pulsating signal) to the DC component (𝐼_𝐷𝐶_, representing the stationary signal) for measurements taken at two distinct wavelengths. The measurement data can be utilized to derive it through the following equation:

$R=\frac{I_{AC(Red)}/I_{DC(Red)}}{I_{AC(IR)}/I_{DC(IR)}}$, $I_{AC}=I_{valley}$, $I_{DC}={I_{peak}-I}_{valley}$

**Encapsulation and sealing test:**

The device is encapsulated with PDMS to reduce the potential reactions with foreign matter. Combine 5 g of PDMS liquid A with 0.5 g of liquid B to prepare the encapsulation solution. Apply this solution to both sides of the device and cure it at 70 °C for one hour to form a robust encapsulation layer. To assess the sealing efficacy of the PDMS encapsulation layer, the photonic devices were submerged in PBS and incubated at 37 °C in an oven for a period of 9 weeks.

**In vivo implantation of biophotonic device:**

Anesthetize the mice using isoflurane anesthesia and disinfect their dorsal surfaces with 75% alcohol. Perform a 2 cm incision using sterile surgical scissors. A PDMS-encapsulated biophotonic device was surgically implanted subcutaneously using forceps, with the sensor precisely positioned adjacent to the femur. The surgical incision was subsequently closed using sutures. The control group was subjected to surgical incisions and pouch creation only, without undergoing device implantation. The mice implanted with devices were positioned on a small radiofrequency plate to monitor the in vivo functionality of the biophotonic device.

**Implantation characterization:**

X-ray imaging: following anesthesia induction with isoflurane, the rat, which had been implanted with a biophotonic device, was transferred to a dynamic flat panel detector for X-ray imaging. The image acquisition system utilizes a-Si image sensor technology, characterized by a frame rate of 40 fps, a pixel size of 85 µm, and an X-ray energy level of 50 keV.

**Thermal characteristics were evaluated as follows:**

The rat implanted with biophotonic device was positioned within a radio frequency cage. Thermal imaging was employed to record temperature changes on the rat’s back under two conditions: with the device turned off and after the device had been operating stably for 10 minutes.

**Behavioral trajectory analysis:**

Conduct a tracking experiment to analyze the behavioral trajectories of SD rats with and without implanted devices utilizing the Animal Behavior Trajectory Video Analysis System V3.0. Place each group of four mice into an open-field recording chamber measuring 25 × 25 × 30 cm, permitting them unrestricted movement. Capture a 20-minute video utilizing a camera mounted above the recording chamber. Subsequently, leveraging advanced computer image processing technology, the behavioral trajectory of the mouse was automatically tracked and recorded. Animal behavior software offers real-time data analysis capabilities, utilizing advanced AI algorithms to display mouse motion parameters, including behavior trajectories, total travel distances, and movement speeds of the tracked subjects.

**Record of sO_2_ levels in freely moving mice:**

Place the SD rats, which have had devices implanted, into a radio frequency transmission cage with a 4 W output. Collect sO_2_ data from skin tissue using biophotonic devices while subjects are in free movement, acquiring red and infrared light signals and sO_2_ data through mobile Bluetooth software.

**Construction of the tumor-bearing model:**

The BALB/c and C57BL/6 mice, aged 6-8 weeks, were purchased from the Laboratory Animal Unit of Xi’an Jiaotong University. 4T1, CT26, and SCC7-based tumor xenograft models were established in mice. Specifically, 4T1 and CT26 cells (3 × 10^7^ cells each) were individually suspended in 200 µL of PBS and subsequently subcutaneously injected into the dorsal regions of BALB/c mice to establish breast and colon cancer models. A skin cancer model was established by subcutaneously injecting SCC7 cells into the dorsal region of C57BL/6 mice using a standardized protocol.

**In vivo evaluation of tumor hypoxia:**

Collect tumors from 4T1 tumor-bearing mice at volumes of approximately 30, 70, 100, 300, 500, and 700 mm³. Perform tissue sectioning followed by anti-HIF-1α immunofluorescence staining to assess the hypoxic status of tumors at various growth stages. Furthermore, the sO_2_ levels of the 4T1 tumor were continuously monitored throughout its growth period. The sO_2_ levels of 4T1, CT26, and SCC7 tumors were compared once the tumor volumes reached approximately 100 mm³. When the 4T1 tumor volume reached approximately 3000 mm³, it was sectioned along the central axis to evaluate sO_2_ levels at different depths within the tumor.

In vivo chemotherapy and sO_2_ evaluation: 4T1 tumor-bearing mice were randomly divided into three groups: (1) PBS, (2) intravenous injection of DMXAA, and (3) intratumoral injection of DMXAA. When the 4T1 tumor reached about 100 mm³, the treatment group received DMXAA (12.5 mg kg^−1^) daily for two days, while the control group received PBS. Photographs of the tumors were taken before and after DMXAA injection, and the sO_2_ levels of each tumor were continuously monitored using the biophotonic device. The tumor volumes were measured bi-daily utilizing the formula V = (length × width²)/2. Following a 6-day treatment period, the tumors were harvested and subjected to H&E staining for the evaluation of pathological changes. Additionally, immunofluorescence staining of CD31 and HIF-1α was also performed to assess the extent of tumor blood vessel destruction and hypoxic conditions.

**Synthesis of Ce6-F127:**

The photosensitizer Ce6 was selected for its compatibility with the 660 nm μ-LED component of the biophotonic device. Ce6 and F127 were self-assembled into Ce6-F127 nanoparticles to improve the limited aqueous solubility of Ce6. The preparation of Ce6-F127 was conducted as follows: 5 mg of Ce6 and 15 mg of F127 were dissolved in DMSO; then the resulting solution was dialyzed in a dialysis bag with a molecular weight cutoff of 1000 Da for 24 hours to facilitate self-assembly, yielding a yellow-green aqueous solution of Ce6-F127 nanoparticles.

Photodynamic performance of Ce6-F127: The capability of Ce6-F127 to generate ROS was measured using DPBF. 20 μL of DPBF solution (DMSO, 1.5 mg mL^−1^) was added to 2 mL of Ce6-F127 solution (DMSO, 10 μg mL^−1^), and then irradiated by a 660 nm LED with 4.5 mW cm^−2^. Afterwards, the absorption intensity at 416 nm was collected at two-minute intervals and plotted as a function of irradiation time.

**In vitro photodynamic therapy:**

The 4T1 cells were seeded at a density of 5 × 10^3^ cells per well in a 96-well plate and incubated for 24 hours under standard culture conditions. The cells were co-incubated with Ce6-F127 at a concentration of 5 μg mL^−1^ for an additional 6 hours, after which they were irradiated with a 660 nm LED (4.5 mW cm^−2^) for a duration of 12 hours. Cell viability was evaluated using alamarBlue and LIVE/DEAD assays. Untreated 4T1 cells were used as the control group.

**In vivo photodynamic therapy and sO_2_ evaluation:**

The 4T1 tumor-bearing mice were divided into four groups: (1) PBS, (2) intratumoral injection of Ce6-F127, (3) illumination with the biophotonic device, and (4) combination treatment of Ce6-F127 and illumination with the biophotonic device. When the tumor volumes reached approximately 100 mm³, mice were anesthetized using isoflurane. A 2-cm incision was made on the dorsal surface of each mouse for the implantation of a photon device. The sensor component of the device was positioned beneath the tumor, after which the incision was closed with sutures. Following intratumoral administration of Ce6-F127 (100 μg mL^−1^, 0.5 mL), the biodistribution of Ce6-F127 within murine tumors and major organs was evaluated using an in vivo imaging system. To investigate the impact of varying laser powers on O_2_ consumption during the PDT process, a 660 nm laser was employed as the light source. The sO_2_ levels of tumor were examined by the biophotonic device, following three cycles of 20 minutes of light irradiation and 20 minutes of darkness at power densities of 4.5, 60, 100, and 200 mW cm^−2^, respectively. Furthermore, the μ-LED on the device was utilized to irradiate the tumor for 1 hour at intervals of 1 hour, with a total of 5 cycles conducted. Throughout the intermittent light treatment spanning 10 hours, continuous monitoring and collection of tumor sO_2_ data were performed. Intermittent PDT was administered to the tumor on days 1 through 3, with a cumulative treatment duration of 12 hours per day. Tumor dimensions were evaluated at two-day intervals. Following a 10-day treatment period, the mice were humanely euthanized, and tumor tissues were harvested for further analysis. The apoptosis and proliferation of 4T1 cells were assessed using H&E staining and Ki-67 immunohistochemical staining, respectively. Additionally, hypoxic conditions within the tumor microenvironment were evaluated via HIF-1α immunofluorescence staining.

**Code availability:**

The codes that support the findings of this study are available as following: https://github.com/13287388612/Biophotonic-device.git.

**Statistical analysis:**

The GraphPad Prism software (version 9.0) and ImageJ software (version 2.1.0) were used for the statistical analysis. All data were obtained from at least three parallel samples per condition in each experiment and are expressed as mean ± standard deviations (SD). Multiple comparisons were performed using one-way two-sided analysis of variance (ANOVA) with Tukey’s multiple comparison test. A probability value of p < 0.05 was considered statistically significant (**p* < 0.05, ***p* < 0.01, ****p* < 0.001, *****p* < 0.0001).

**Animal experiment approval:**

The animal experiments studies were conducted in accordance with the guidelines of the Administration of Laboratory Animals of China and approved by the animal ethics committee of Northwestern Polytechnical University (Number: NPU202201018).

**Supplementary Figures and Tables**


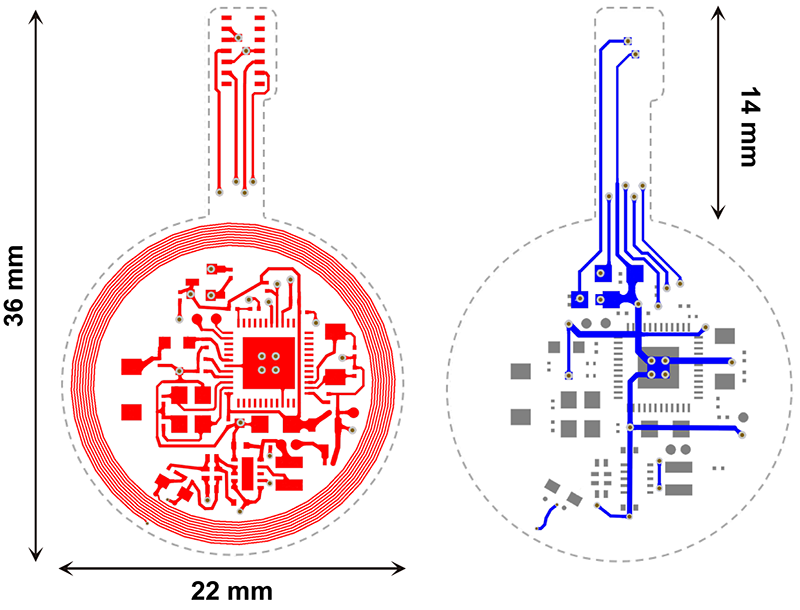


**Figure S1.** Layout of printed circuit boards for devices: red for the top layer, blue for the bottom layer.


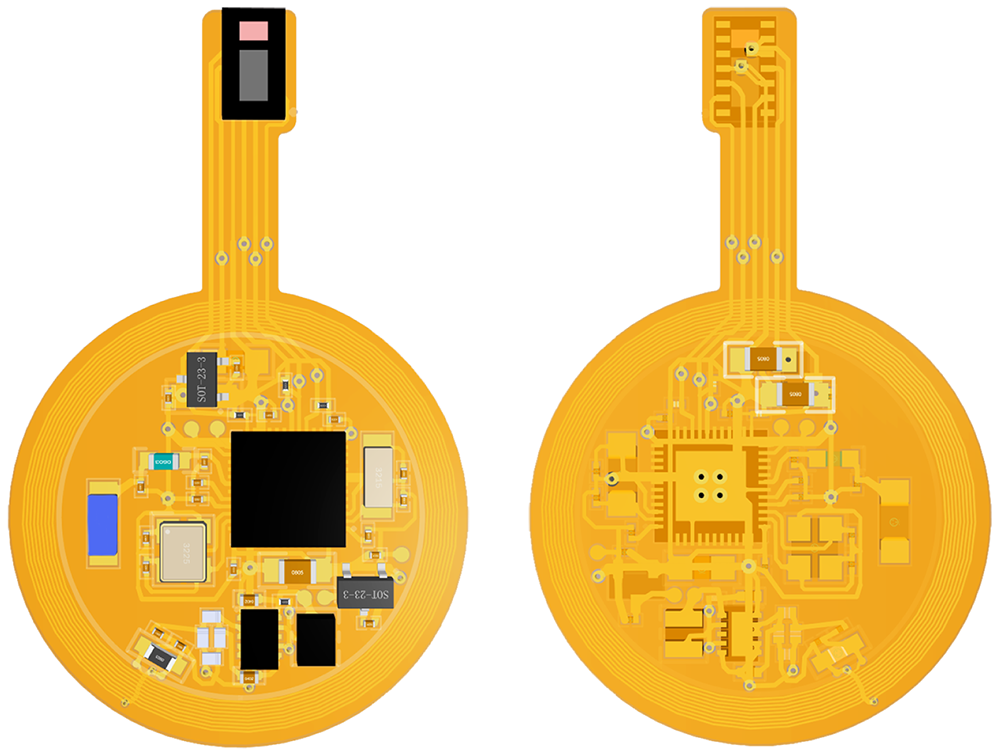


**Figure S2.** 3D model diagram of the device.


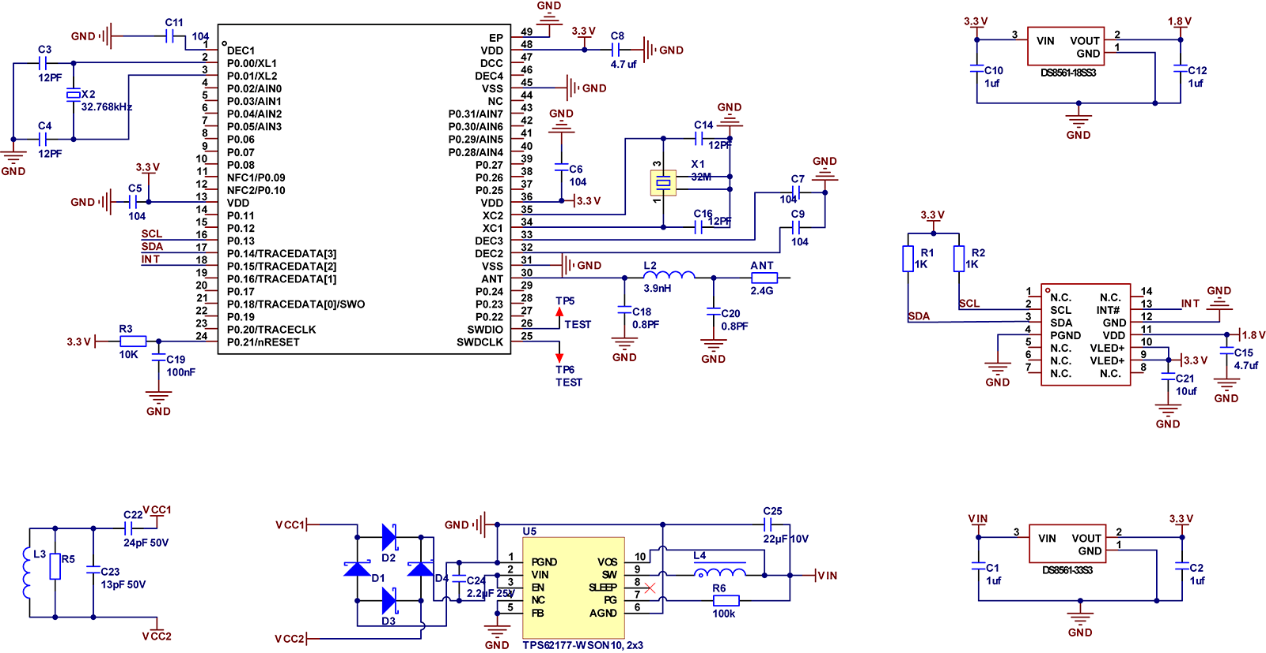


**Figure S3.** Detailed circuit design diagram of the device.


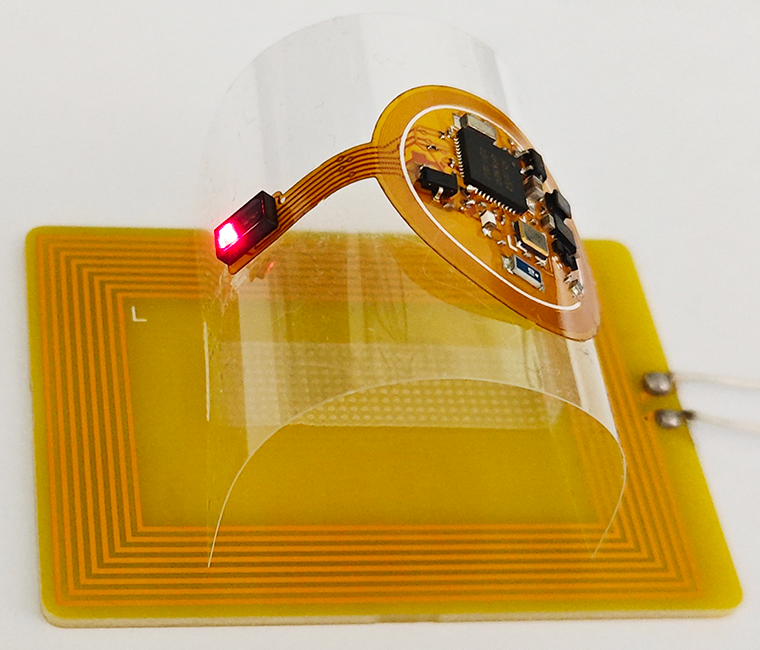


**Figure S4.** Photo of the device during bending operation with a 15-mm bending radius. RF power input to the transmitting antenna board is 4 W.


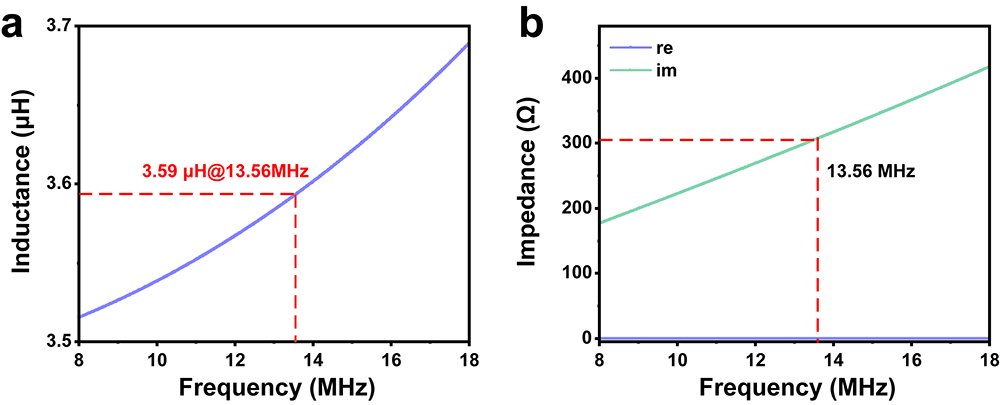


**Figure S5.** The finite element analysis (FEA) results of the wireless receiving coil characteristics for photonic devices include a) inductance and b) impedance.


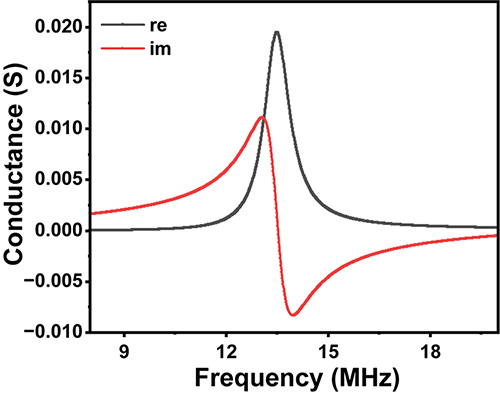


**Figure S6.** The frequency-dependent curves of the real and imaginary parts of the admittance for a photonic device's wireless receiving resonant circuit.


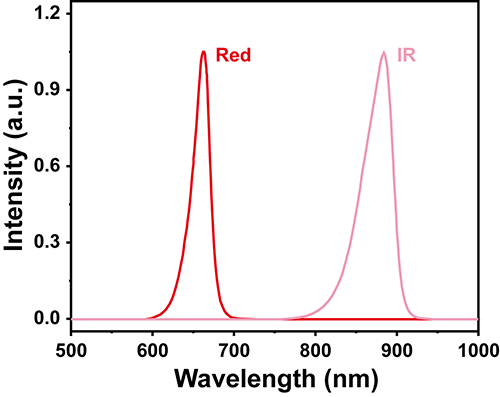


**Figure S7.** The red and NIR LED emission spectral characteristics of the MAX30102 sensor: red LED: 660 nm; NIR LED: 880 nm.


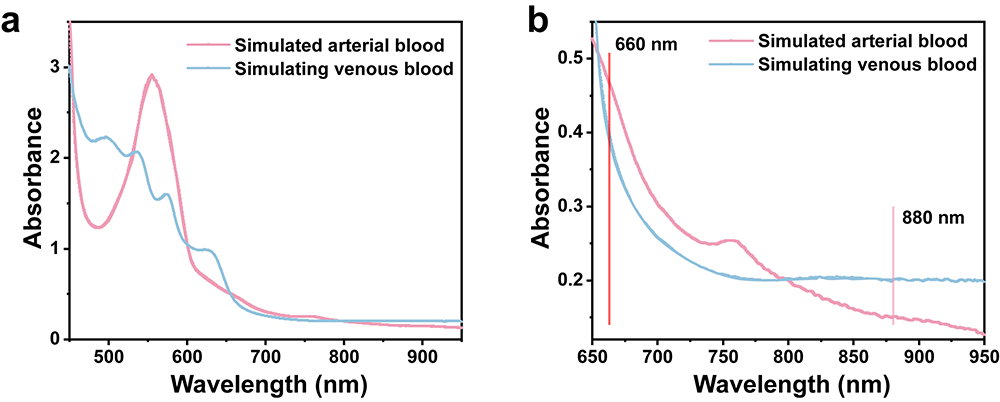


**Figure S8.** Artificial blood UV absorption spectrum. Full UV-vis absorption spectroscopy a) and localized amplification absorption spectroscopy b) of simulated arterial blood and simulated venous blood.


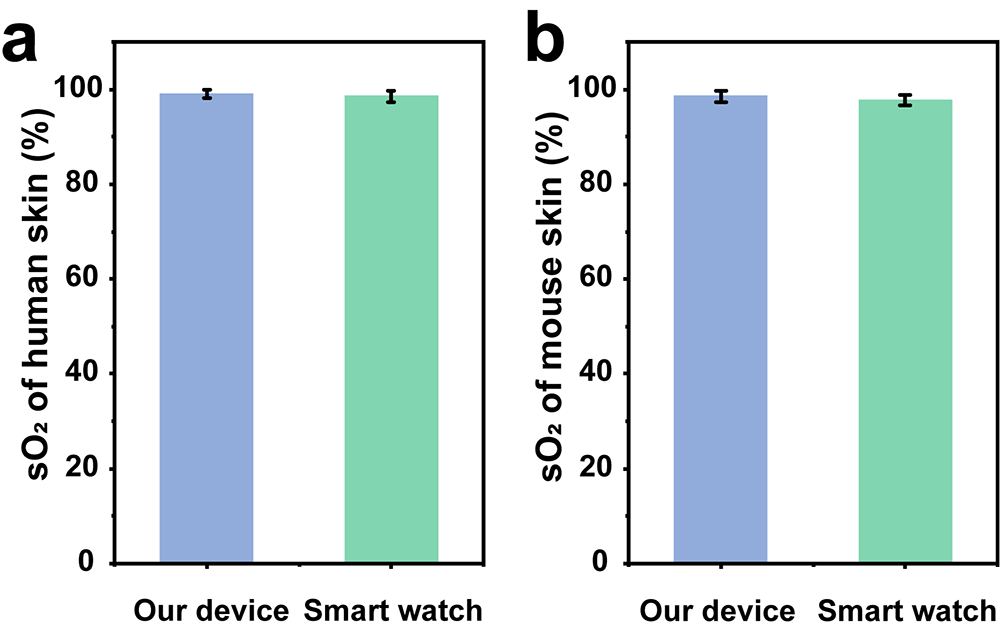


**Figure S9.** sO_2_ values of human skin a) and mouse skin b) were measured by devices and Xiaomi smartwatches.


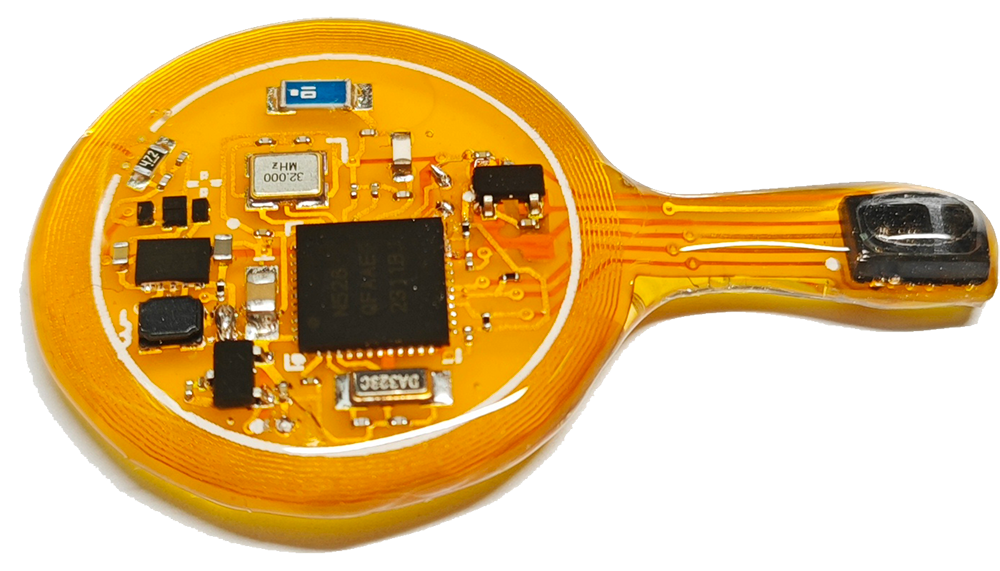


**Figure S10.** Photos of PDMS-packaged devices.


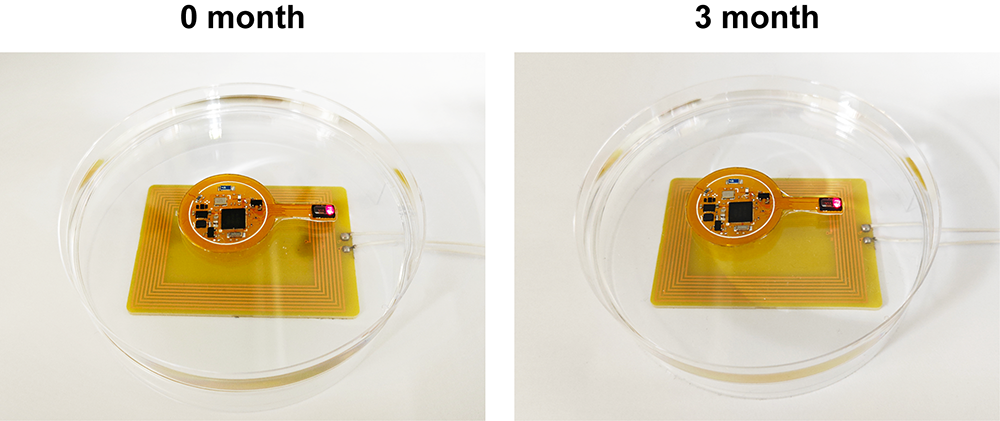


**Figure S11.** Sealing test of PDMS-encapsulated photonic devices: the devices function normally after 3 months in PBS solution, as shown in the photos.


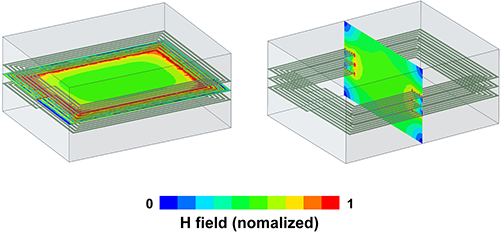


**Figure S12.** Simulate the normalized magnetic field distribution inside the wireless power supply RF transmission cage in both transverse and longitudinal cross-sections.


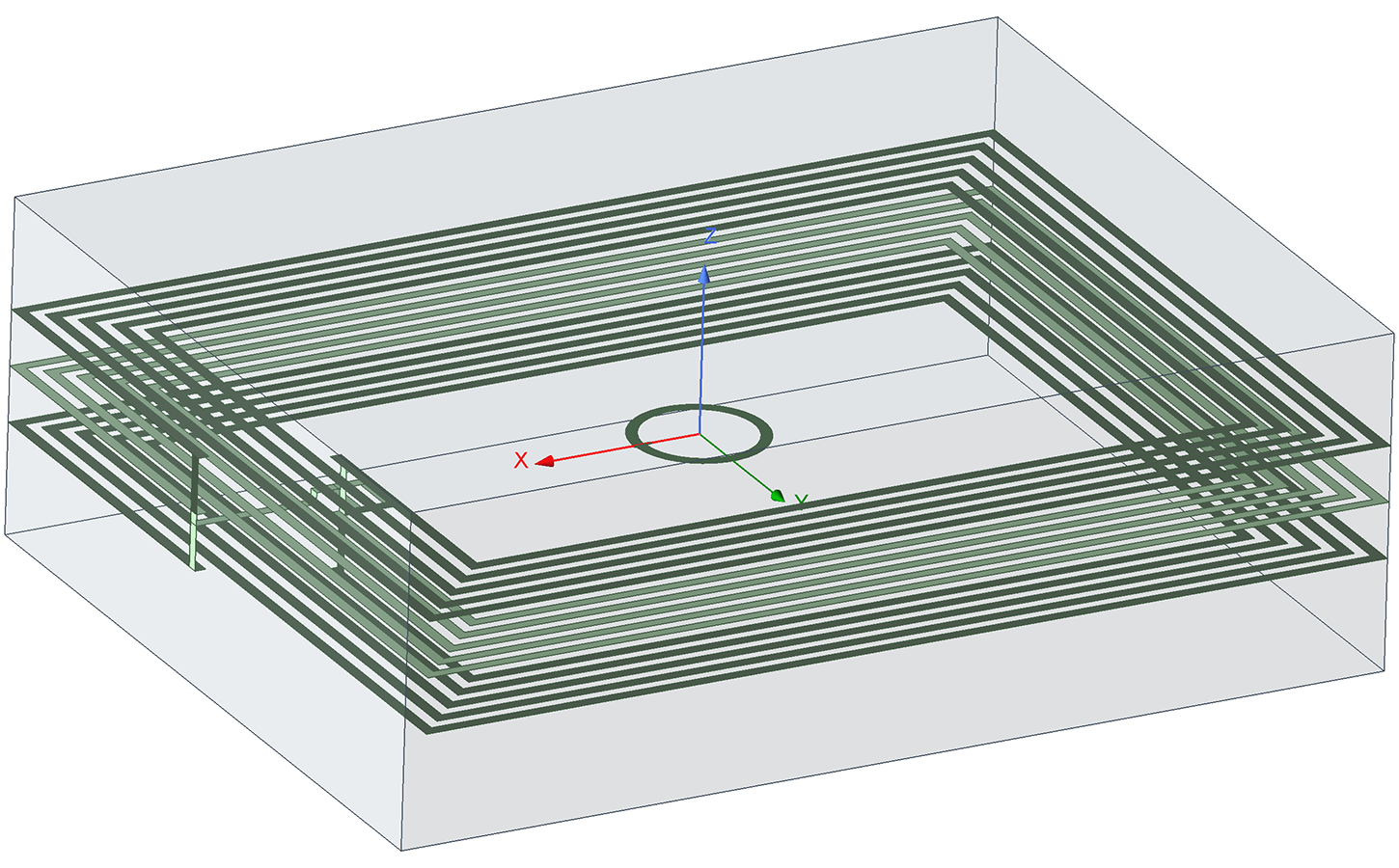


**Figure S13.** Schematic diagram of the FEA of the wireless power transmission (WPT) system structure: The RX coil is positioned at the center of the TX resonant coil.


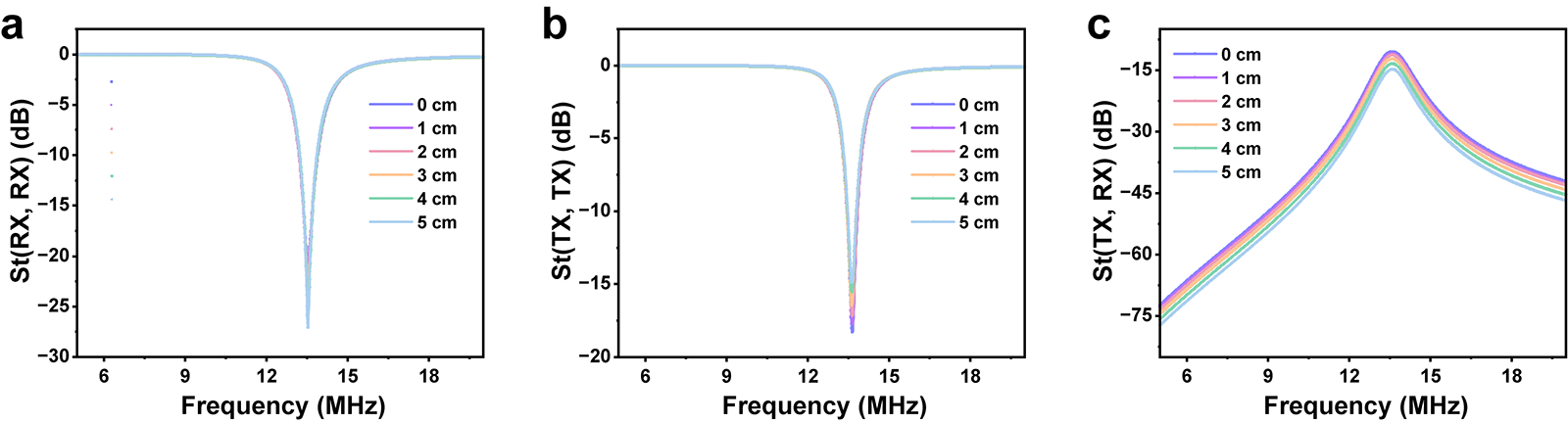


**Figure S14.** Perform FEA of the relationship curves between energy transmission loss parameters a) S(RX, RX) and b) S(TX, TX), as well as the gain parameter c) S(TX, RX) versus frequency variation. Place the RX resonant coil of the photonic device along the central axis of the TX resonant coil, at a distance of 0-5 cm from the center of the TX coil.


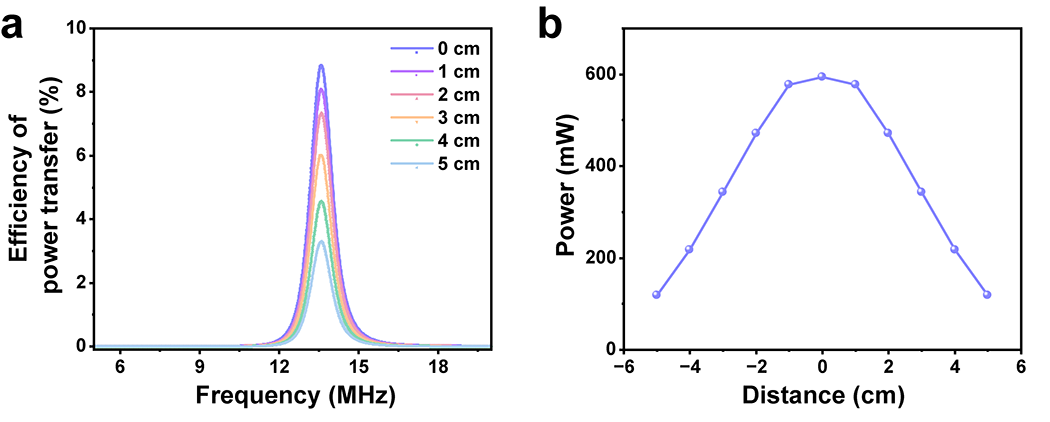


**Figure S15.** a) FEA models the relationship between WPT efficiency and frequency at various distances. b) The curve shows the variation of the wireless transmission power test values received by the receiving coil with the transmission distance. The transmitting coil has an input power of 4 W, and the resonant coil is connected to a 1 kΩ load.


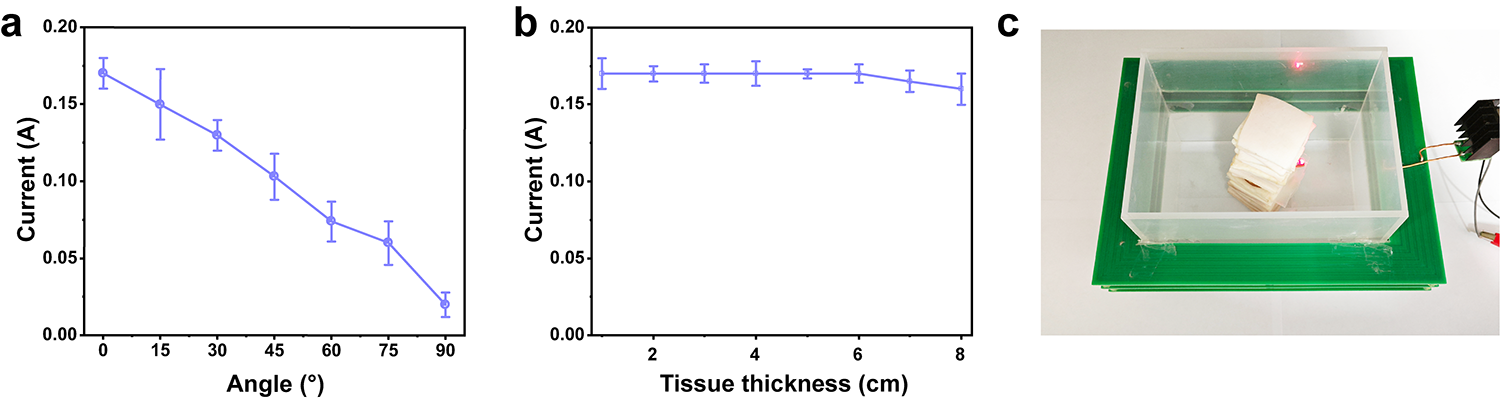


**Figure S16.** The current values received by the receiving coil and power management module (connected to a 1 kΩ load, with a constant output voltage of 3.3 V) of the device at different angles a) or when buried in tissues of different thicknesses b) in the wireless power transmission cage. The transmitting coil has an input power of 4 W, and the resonant coil is connected to a 1 kΩ load.


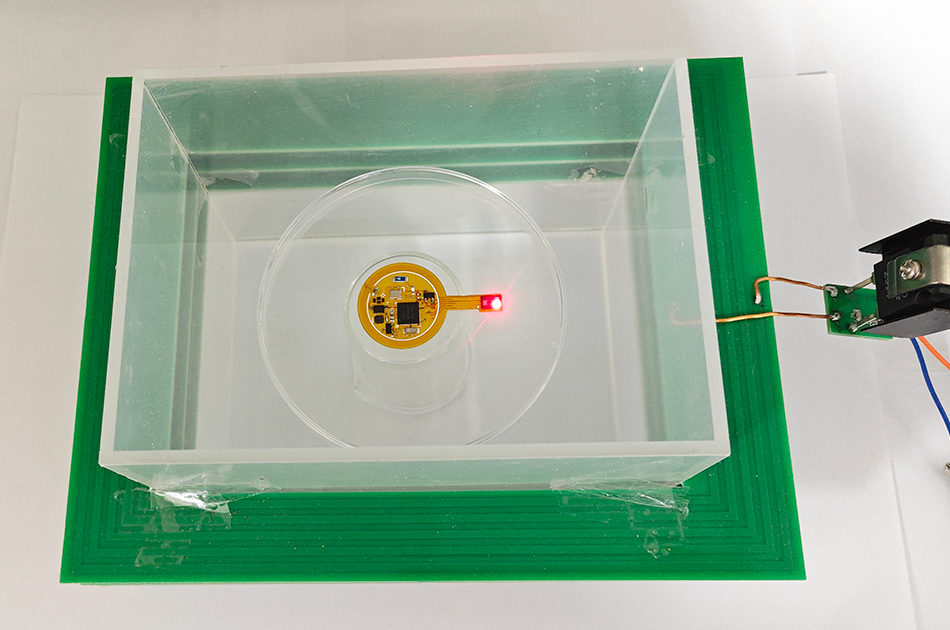


**Figure S17.** Photo of the device operating normally in a wireless RF transmission chamber.


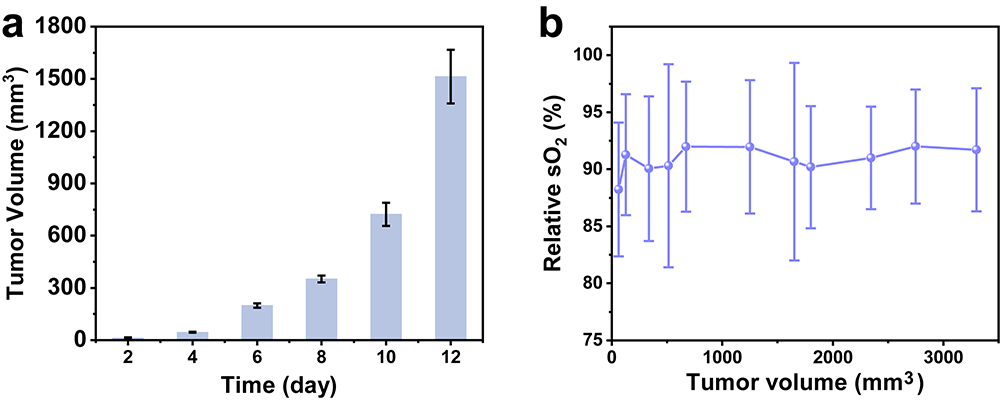


**Figure S18.** Changes in tumor volume and sO_2_ during growth. a) Statistical chart of tumor growth volume from day 1 to day 12. b) The volume-dependent curve of tumor sO_2_.


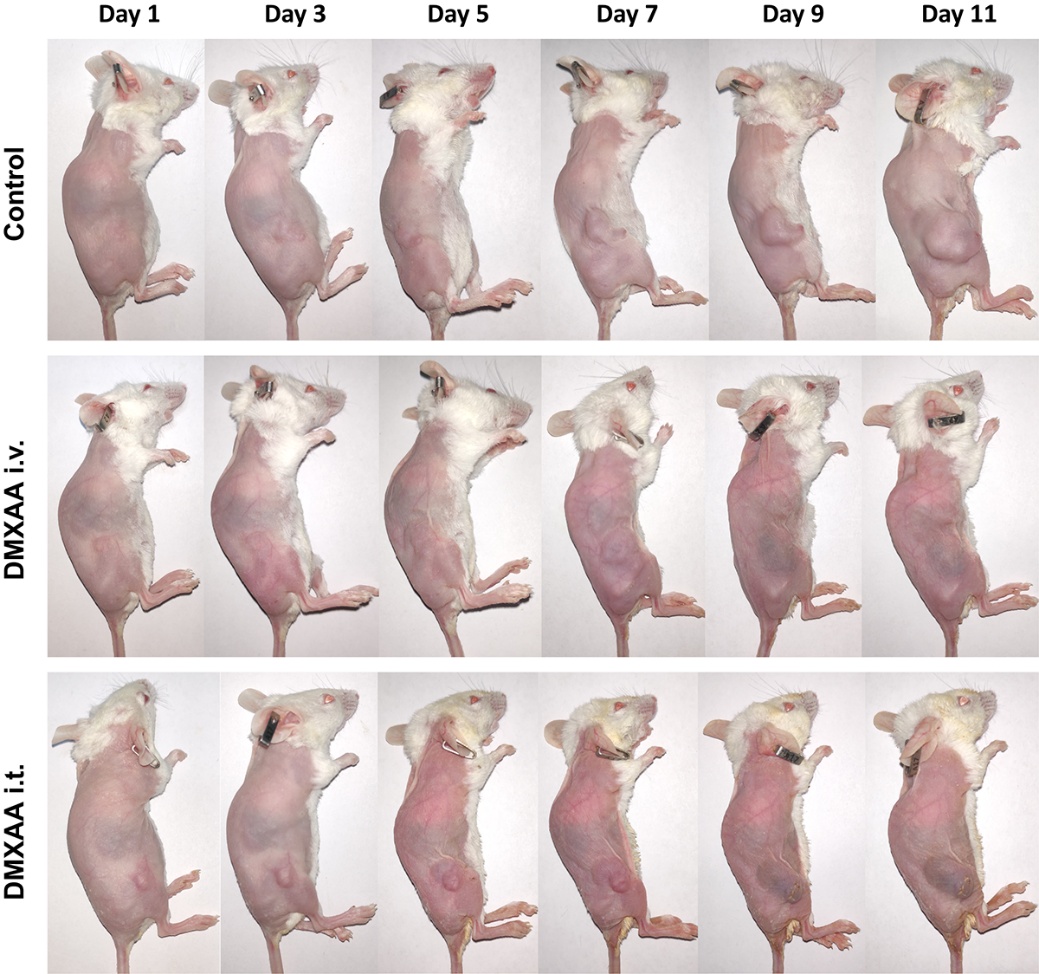


**Figure S19.** Tumor images of mice from different treatment groups before and after DMXAA injection in a tumor vascular disruption model.


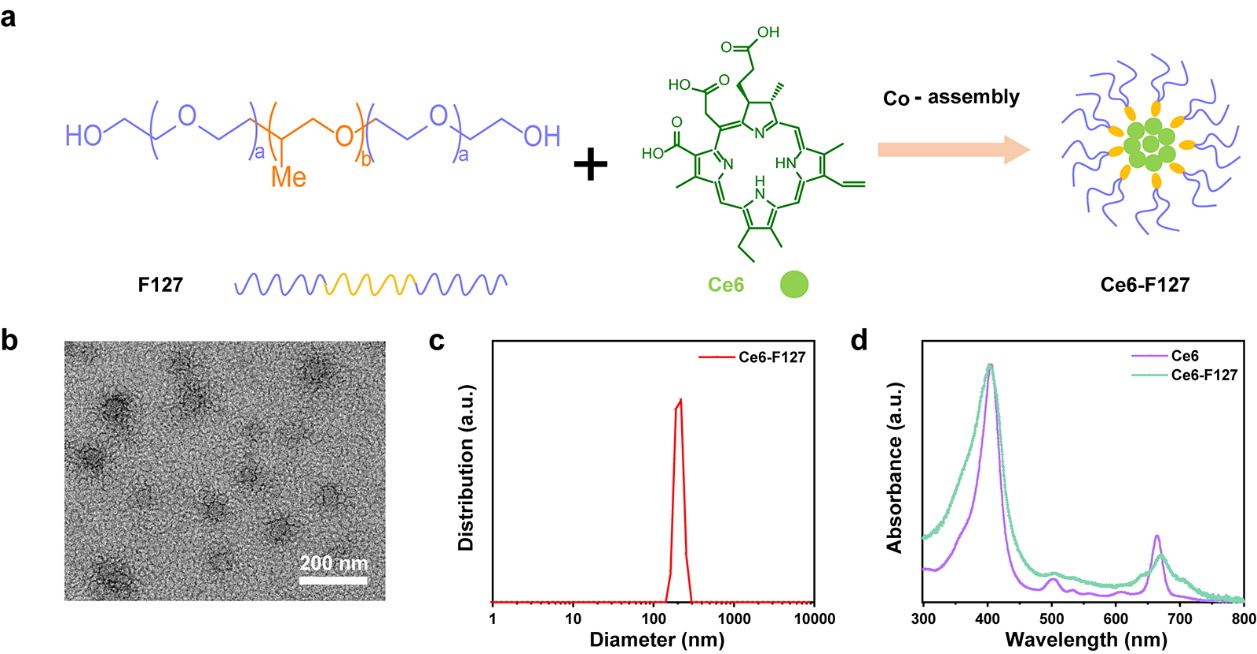


**Figure S20.** Synthesis and characterization of Ce6-F127. a) Synthesis mechanism diagram. b) TEM image of Ce6-F127 assembly. c) Particle size distribution of Ce6-F127 hydration. d) UV-Vis absorption spectra of Ce6 and Ce6-F127.


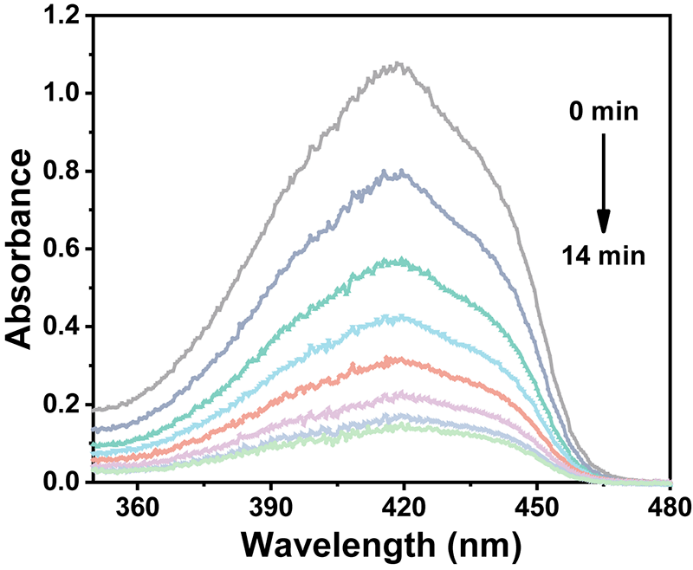


**Figure S21.** Characterization of photo-induced ROS generation performance of Ce6-F127: UV-Visible absorption spectroscopy of DPBF photodegraded by Ce6-F127. The light source is a 660 nm LED with a power of 4.5 mW cm^-^².


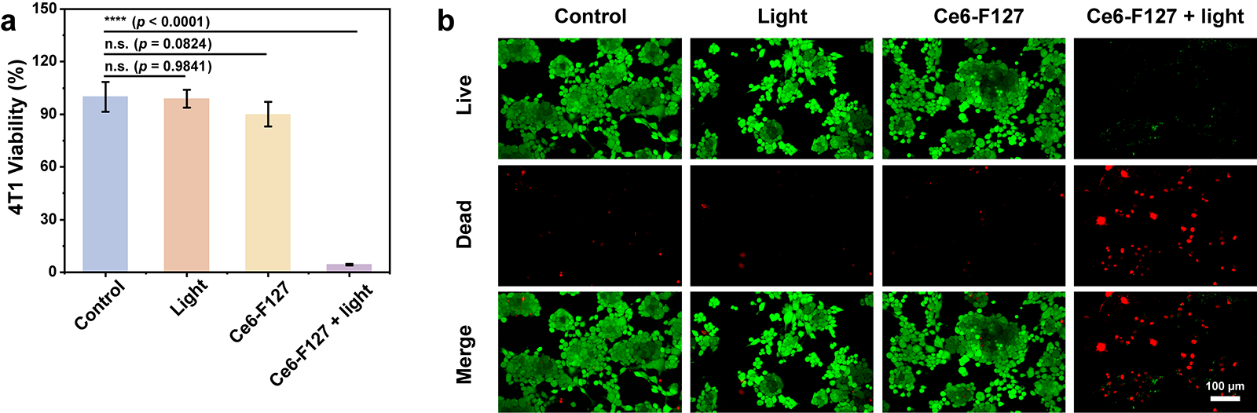


**Figure S22.** Ce6-F127 kills tumor cells through PDT. a) Cell survival rate bar chart and b) Live/Dead cell staining image. The light source is a 660 nm LED with a power of 4.5 mW cm^-^².


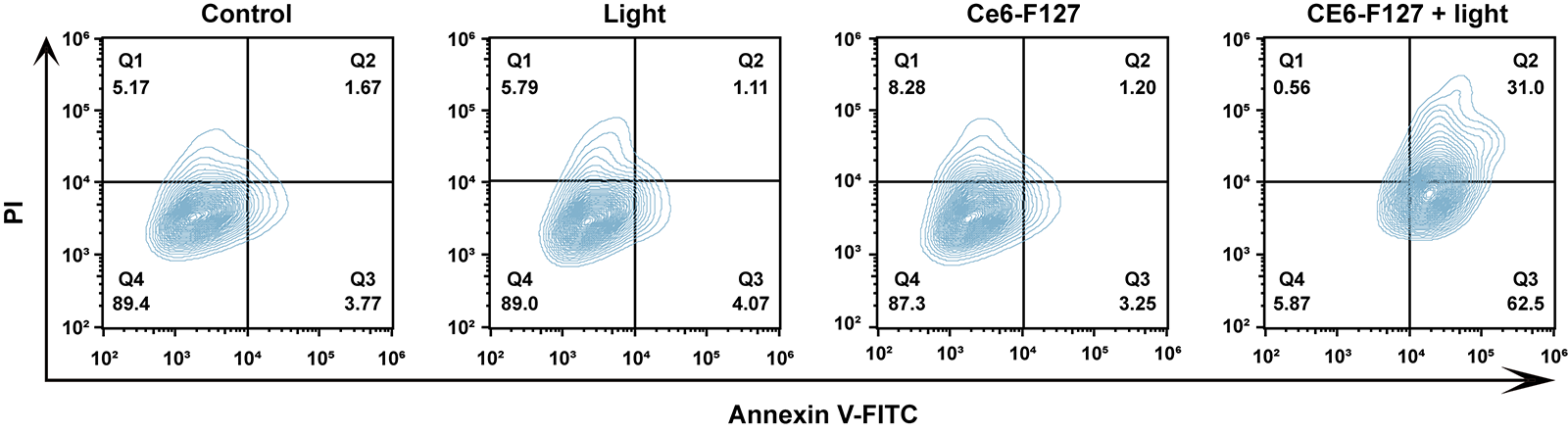


**Figure S23**. Flow cytometry analysis of V-FITC/PI stained 4T1 cells after different treatments.


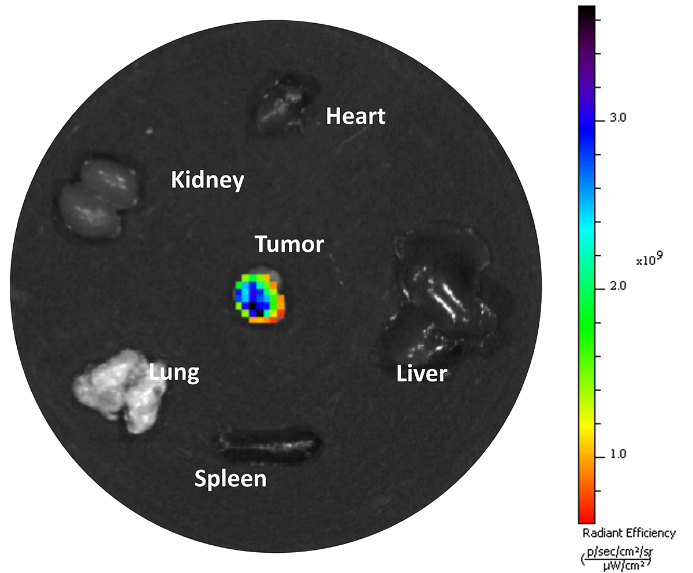


**Figure S24.** After intratumoral injection, the distribution of Ce6-F127 in mouse tumors and major organs (heart, liver, spleen, lungs, kidneys) was monitored using a small animal live imaging system.


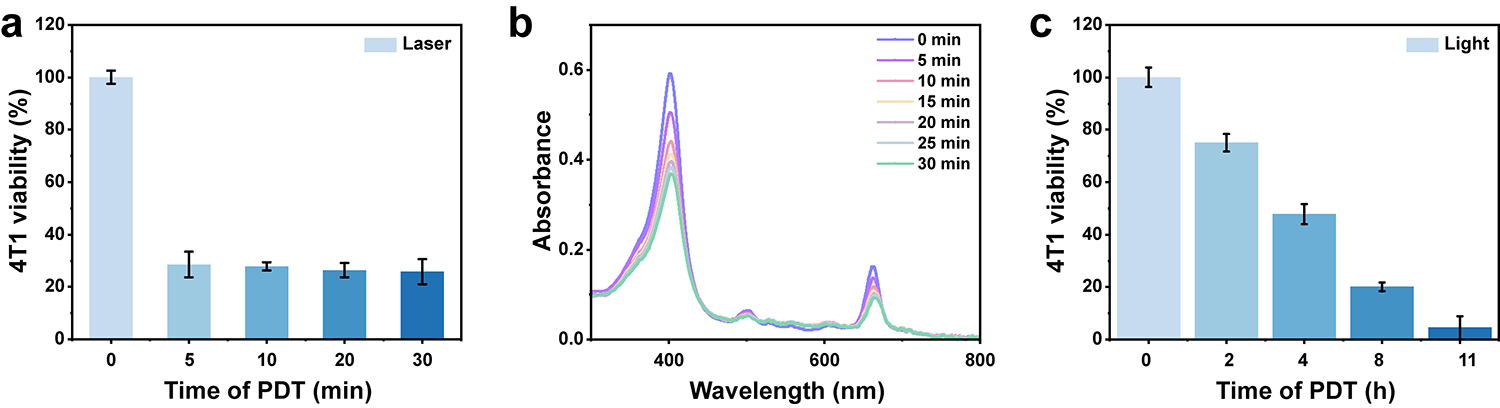


**Figure S25**. a) The survival rate of 4T1 cells co-cultured with Ce6 (5 μg ml⁻¹) following exposure to a 660 nm laser (100 mW cm⁻²) at various irradiation durations. b) The absorption spectrum of Ce6 after being irradiated by 660 nm laser (100 mW cm^-2^) for varying durations. c) The survival rate of 4T1 tumor cells co-cultured with Ce6 (5 μg ml⁻¹) after exposure to LED light (4.5 mW cm⁻²) for different periods of time.


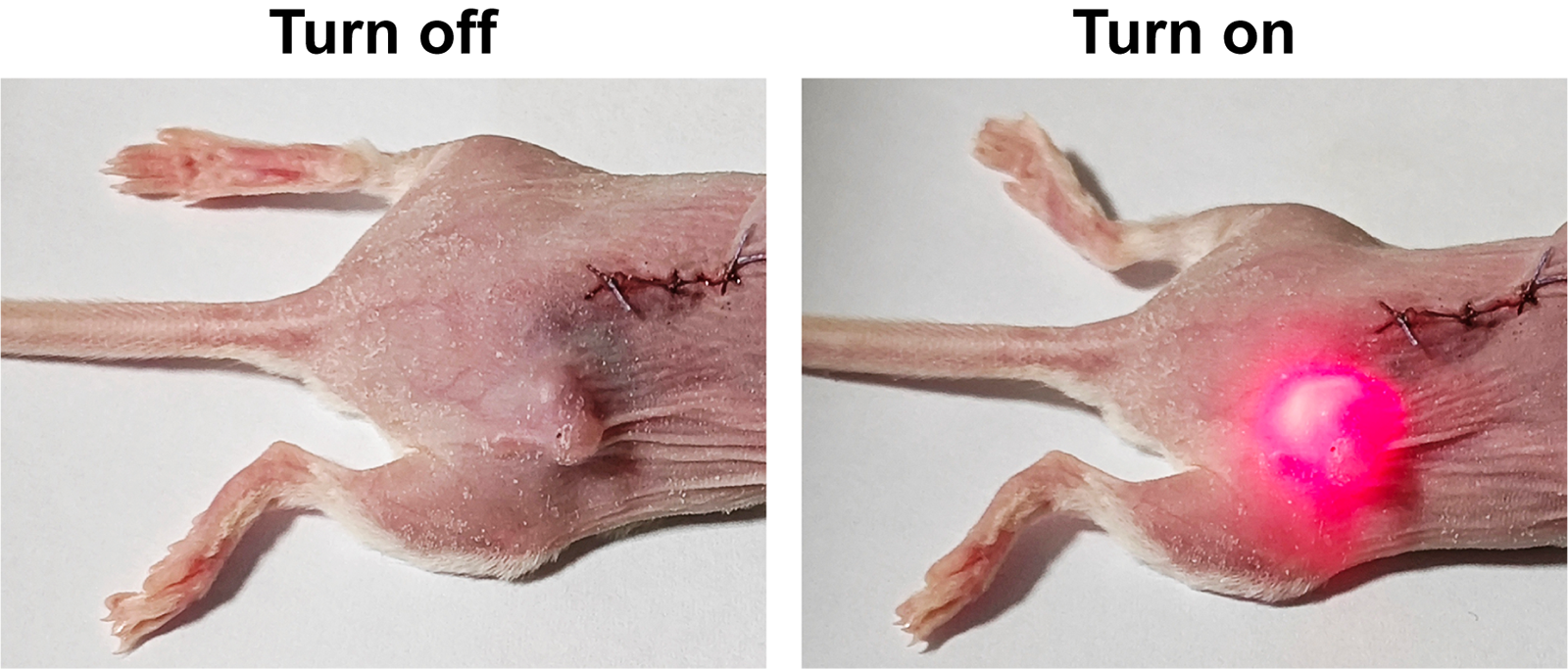


**Figure S26.** Photo of photodynamic therapy in mice implanted with optical devices.


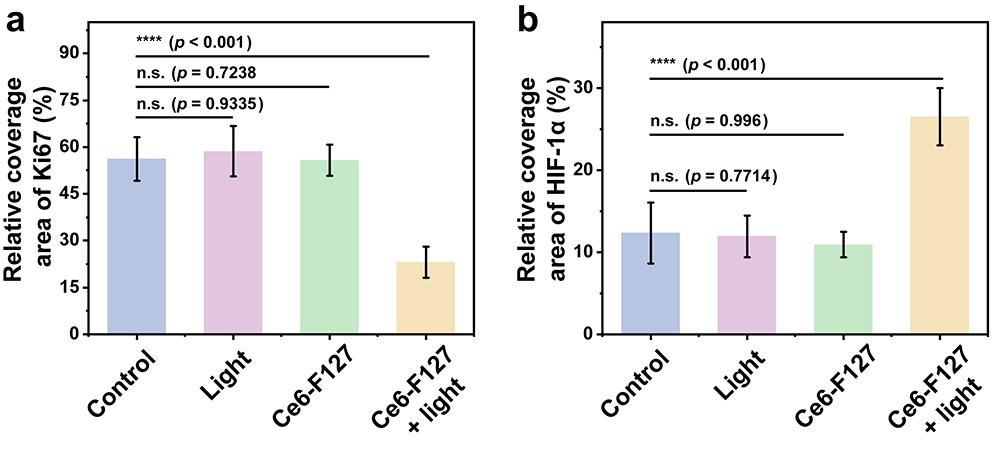


**Figure S27.** Quantitative statistical chart of a) Ki67 and b) HIF-1α staining in tumor tissue post-photodynamic therapy.


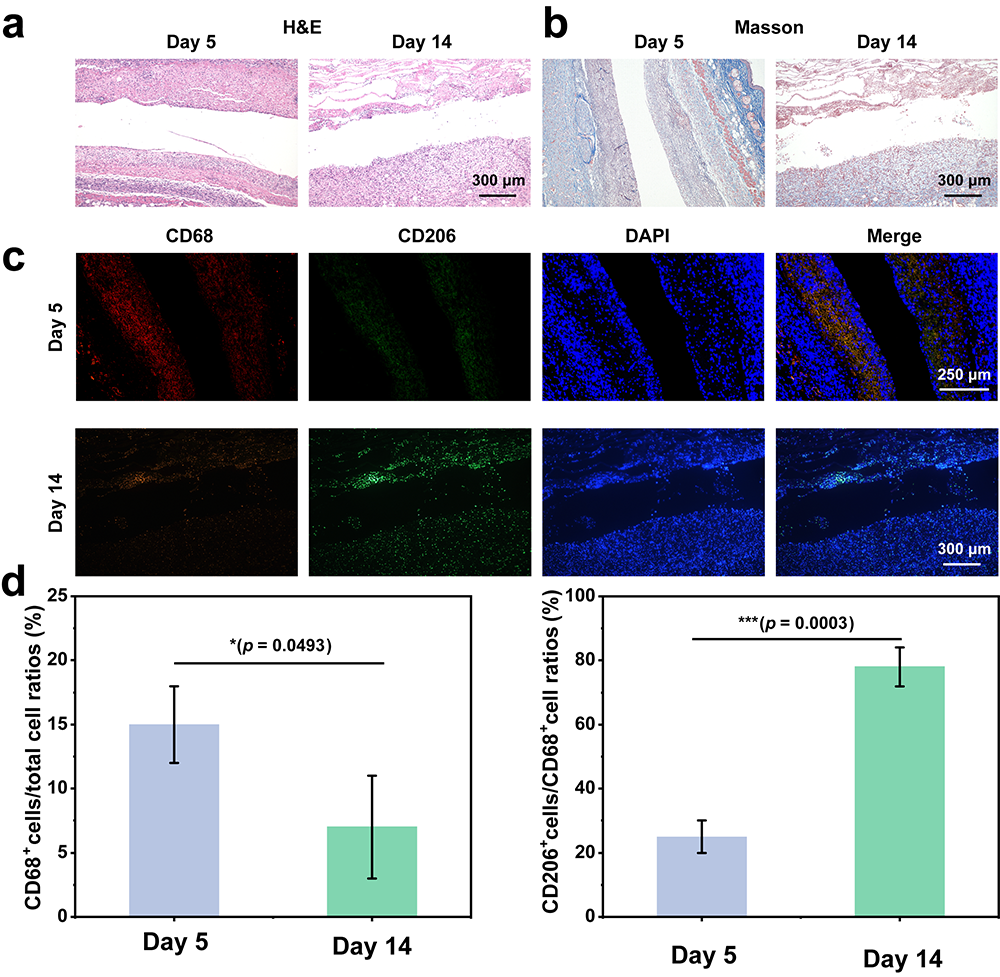


**Figure S28**. Biocompatibility evaluation of the device implanted in mice. a) H&E staining of surrounding tissues on post-subcutaneous implantation days 5 and 15. b) Masson trichrome staining of surrounding tissues on post-subcutaneous implantation days 5 and 15. c) Immunofluorescence staining images of M1 macrophages (CD68) and M2 macrophages (CD206). d) Statistical quantification of the proportion of CD68-positive cells among total cells and the ratio of CD206 positive cells to CD68 positive cells.

| **Symbol** | **Component** | **Package** |
| --- | --- | --- |
| ANT | 2.4 G | OSC 3215-2P |
| C1, C2, C10, C12 | Capacitor, 1 μf | C 0201 |
| C3, C4, C14 | Capacitor, 12 pF | C 0201 |
| C5, C6, C7, C9，C11 | Capacitor, 0.1 μf | C 0201 |
| C8, C15 | Capacitor, 4.7 μf | C 0805 |
| C16 | Capacitor, 12 pF | C 0201 |
| C18，C20 | Capacitor, 0.8 pF | C 0201 |
| C19 | Capacitor, 100 nF | C 0201 |
| C21 | Capacitor, 10 μf | C 0805 |
| C22 | Capacitor, 24 pF 50 V | C 0201 |
| C23 | Capacitor, 13 pF 50 V | C 0201 |
| C24 | Capacitor, 2.2 μF 25 V | C 0402 |
| C25 | Capacitor, 22 μF 10 V | C 0402 |
| D1, D2, D3, D4 | Schottky diode, PMEG3010AESBYL, 30 V, 1 A | DSN1006-2 (SOD993) |
| L2 | Inductor, 3.9 nH | L 0603 |
| L4 | Inductor, 10 μH | NR2520, SMD, 2 × 2.5 mm |
| R1, R2 | Resistor, 1 K | R 0201 |
| R3 | Resistor, 10 K | R 0201 |
| R5 | Resistor, 4.7 K, 0.1 W | R 0603 |
| R6 | Resistor, 100 k | R 0201 |
| U1 | NRF52832-QFAA-T | QFN-48, 6 × 6 mm |
| U2 | LDO, DS8561-33S3 | SSOT-23-3 |
| U3 | LDO, DS8561-18SS3 | SSOT-23-3 |
| U4 | MAX30102EFD+T | OESIP-14 |
| U5 | DC-DC, TPS62177 | WSON10, 2 × 3 mm |
| X1 | XTAL, 32 MHz | OSC 3225-4P |
| X2 | XTAL, 32.768 kHz | OSC 3215-2P |

**Table S1.** List of components used in photonic device circuit systems.

**Table S2.** Comparing the functions and principles of implantable and wearable photonic diagnostic and therapeutic devices reported in the literature and this work.

| **Ref.** | **Function** | **Diagnosis principles** | **Treatment principle** | **Energy supply method** | **Operation method** |
| --- | --- | --- | --- | --- | --- |
| 19 | Tumor identification | Diffuse reflection of light | None | None | Operation |
| 20 | Tumor identification | Fiber optic endoscope | None | None | Operation |
| 21 | Treatment | None | PDT | Piezoelectric effect | Implantable |
| 22 | Treatment | None | PDT | PENG | Wearable |
| 23 | Treatment | None | PDT/SDT | Piezoelectric effect | Implantable |
| 24 | Treatment | None | PDT | WPT | Implantable |
| 25 | Treatment | None | PTT | WPT | Implantable |
| 26 | Treatment | None | PDT | TENG | Wearable |
| 27 | Tumor identification and treatment | Refraction of light | PTT | Wired | Implantable |
| 28 | Tumor identification and treatment | Hypoxia-sensitive fluorescent probes | PTT | Wired | Implantable |
| 29 | Tumor identification and treatment | Fluorescence imaging | Laser ablation | Wired | External equipment |
| 30 | Tumor identification and treatment | Fluorescence imaging | PTT | None | Implantable |
| 31 | Monitoring and treatment | Diffuse reflection of light | PDT | WPT | Insert tumor |
| This work | Diagnosis, Monitoring and treatment | sO_2_ monitoring | PDT | WPT | Implantable/Wearable |

Video S1 (separate file): Sealing test of PDMS-encapsulated photonic devices.

Video S2 (separate file): Mice implanted with devices move freely in RF transmission cages.

**References**

[19] S. Y. Lee, J. M Pakela, K. Na, J. Shi, B. J McKenna, D. M Simeone, E. Yoon, J. M Scheiman, M. Mycek, Needle-compatible miniaturized optoelectronic sensor for pancreatic cancer detection. *Sci. Adv.* **2020**, *6*, eabc1746.

[20] E. Pshenay-Severin, H. Bae, K. Reichwald, G. Matz, J. Bierlich, J. Kobelke, A. Lorenz, A. Schwuchow, T. Meyer-Zedler, M. Schmitt, B. Messerschmidt, J. Popp, Multimodal nonlinear endomicroscopic imaging probe using a double-core double-clad fiber and focus-combining micro-optical concept. *Light Sci. Appl.* **2021**, *10*, 207.

[21] Q. Han, Z. Fang, R. Lin, J. Chen, X. Wei, C. Gong, Z. Yang, P. Zou, J. Zhu, L. Xing, X. Xue, J. Lang, Y. Zhou, M. Chen, Piezo-photodynamic therapy of Au@PEG-ZnO nanostructures enabled with a battery-free wireless cancer therapeutic dot. *Nano Energy* **2024**, *125*, 109530.

[22] T. Lin, P. Zou, R. Lin, H. Guan, Z. Fang, J. Chen, Z. Long, Y. Zhang, L. Xing, F. Qi, J. Lang, X. Xue, M. Chen, A self-powered wireless detachable drug/light injector for metronomic photodynamic therapy in cancer treatment. *Nano Energy* **2023**, *116*, 108826.

[23] H. Guan, P. Zou, R. Lin, L. Xiao, Z. Fang, J. Chen, T. Lin, Y. Wang, Y. Peng, T. Zhong, B. Zhang, J. Lang, Y. Zhang, L. Xing, M. Chen, X. Xue, Implantable self-powered therapeutic pellet for wireless photodynamic / sonodynamic hybrid therapy of cancer recurrence inhibition and tumor regression. *Nano Energy* **2023**, *105*, 108002.

[24] K. Yamagishi, I. Kirino, I. Takahashi, H. Amano, S. Takeoka, Y. Morimoto, T. Fujie, Tissue−adhesive wirelessly powered optoelectronic device for metronomic photodynamic cancer therapy. *Nat. Biomed. Eng.* **2019**, *3*, 27.

[25] H. Arami, S. Kananian, L. Khalifehzadeh, C. B Patel, E. Chang, Y. Tanabe, Y. Zeng, S. J Madsen, M. J Mandella, A. Natarajan, E. E Peterson, R. Sinclair, A. S Y Poon, S. S. Gambhir, Remotely controlled near-infrared-triggered photothermal treatment of brain tumours in freely behaving mice using gold nanostars. *Nat. Nanotechnol*. **2022**, *17*, 1015.

[26] Z. Liu, L. Xu, Q. Zheng, Y. Kang, B. Shi, D. Jiang, H. Li, X. Qu, Y. Fan, Z. L. Wang, Z. Li, Human Motion Driven Self-powered photodynamic system for long-term autonomous cancer therapy. *ACS Nano* **2020**, *14*, 8074.

[27] H. Wu, P. Chen, X. Zhan, K. Lin, T. Hu, A. Xiao, J. Liang, Y. Huang, Y. Huang, B. Guan, Marriage of a Dual-plasmonic interface and optical microfiber for NIR-II cancer theranostics. *Adv. Mater* **2023**, *36*, 2310571.

[28] Y. Ran, Z. Xu, M. Chen, W. Wang, Y. Wu, J. Cai, J. Long, Z. Chen, D. Zhang, B. Guan, Fiber-optic theranostics (FOT): interstitial fiber-optic needles for cancer sensing and therapy. *Adv. Sci.* **2022**, *9*, 2200456.

[29] Y. Fan, Y. Sun, W. Chang, X. Zhang, J. Tang, L. Zhang, H. Liao, Bioluminescence imaging and two-photon microscopy guided laser ablation of GBM decreases tumor burden. *Theranostics* **2018**, *8*,4072.

[30] S. Geng, P. Guo, J. Wang, Y. Zhang, Y. Shi, X. Li, M. Cao, Y. Song, H. Zhang, Z. Zhang, K. Zhang, H. Song, J. Shi, J. Liu, Ultrasensitive optical detection and elimination of residual microtumors with a postoperative implantable hydrogel sensor for preventing cancer recurrence. *Adv. Mater* **2024**, *36*, 2307923.

[31] K. Kim, I. S. Min, T. H. Kim, D. H. Kim, S. Hwang, K. Kang, K. Kim, S. Park, J. Lee, Y. U. Cho, J. W. Lee, W. Yeo, Y. M. Song, Y. Jung, K. J. Yu, Fully implantable and battery-free wireless optoelectronic system for modulable cancer therapy and real-time monitoring. *npj Flex. Electron*. **2023**, *7*, 41.
